# Exploring the frontiers of condensed-phase chemistry with a general reactive machine learning potential

**DOI:** 10.1038/s41557-023-01427-3

**Published:** 2024-03-07

**Authors:** Shuhao Zhang, Małgorzata Z. Makoś, Ryan B. Jadrich, Elfi Kraka, Kipton Barros, Benjamin T. Nebgen, Sergei Tretiak, Olexandr Isayev, Nicholas Lubbers, Richard A. Messerly, Justin S. Smith

**Affiliations:** 1 Department of Chemistry, Mellon College of Science, Carnegie Mellon University, Pittsburgh, PA USA; 2 Theoretical Division, Los Alamos National Laboratory, Los Alamos, NM USA; 3 Computational and Theoretical Chemistry Group, Department of Chemistry, Southern Methodist University, Dallas, TX USA; 4 Computer, Computational, and Statistical Sciences Division, Los Alamos National Laboratory, Los Alamos, NM USA; 5 Center for Nonlinear Studies, Los Alamos National Laboratory, Los Alamos, NM USA; 6 NVIDIA Corp., Santa Clara, CA USA

**Keywords:** Molecular dynamics, Computational chemistry, Reaction mechanisms

## Abstract

Atomistic simulation has a broad range of applications from drug design to materials discovery. Machine learning interatomic potentials (MLIPs) have become an efficient alternative to computationally expensive ab initio simulations. For this reason, chemistry and materials science would greatly benefit from a general reactive MLIP, that is, an MLIP that is applicable to a broad range of reactive chemistry without the need for refitting. Here we develop a general reactive MLIP (ANI-1xnr) through automated sampling of condensed-phase reactions. ANI-1xnr is then applied to study five distinct systems: carbon solid-phase nucleation, graphene ring formation from acetylene, biofuel additives, combustion of methane and the spontaneous formation of glycine from early earth small molecules. In all studies, ANI-1xnr closely matches experiment (when available) and/or previous studies using traditional model chemistry methods. As such, ANI-1xnr proves to be a highly general reactive MLIP for C, H, N and O elements in the condensed phase, enabling high-throughput in silico reactive chemistry experimentation.

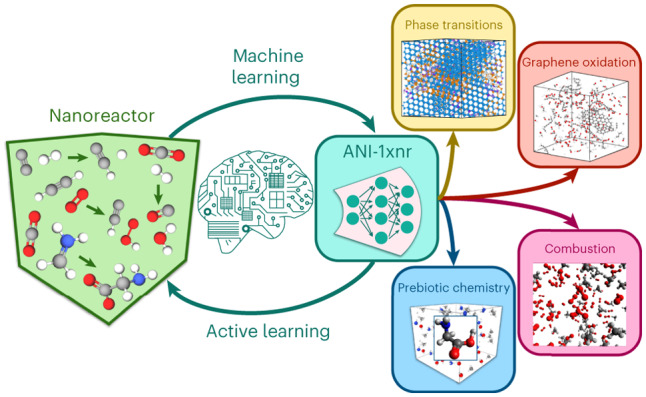

## Abstract (web summary)

Atomistic simulations have a broad range of applications from drug design to materials discovery. Machine learning interatomic potentials (MLIPs) have become an efficient alternative to computationally expensive ab initio simulations. Now a general reactive MLIP (called ANI-1xnr) has been developed and validated against a broad range of condensed-phase reactive systems.

## Main

Over the past several decades, atomic-scale simulation has become an invaluable computational tool for providing microscopic explanations of experimentally observed phenomena. Many scientifically crucial chemical and materials properties can be evaluated through molecular dynamics (MD) simulation, wherein atomic motion is dictated by integrating the second law of Newtonian physics. The quantitative predictiveness of MD depends almost entirely on the accuracy of the underlying model potential energy surface (the potential) used to compute the forces acting on each atom. However, standard physics-based paradigms, such as classical force fields (FFs) and quantum mechanics (QM) methods, straddle a historical trade-off between computational cost, accuracy and generality, that is, being applicable to a broad range of systems without further specialization. This trade-off is especially pronounced in the context of modelling reactions, that is, the making and breaking of chemical bonds. Although computationally efficient, reactive FFs often need to be reparameterized to pre-determined reactions to be quantitatively accurate. By contrast, while QM methods are often quite reliable and generally applicable, their computational cost is prohibitive for many reactive MD studies. For this reason, a fast, accurate and general reactive potential is of paramount importance to many scientific applications, as it would fulfil the long-sought promise for predictive MD simulations that can provide reliable reaction rates, discover entire reaction networks and warn of dangerous conditions, all before entering the laboratory.

Recently, machine learning interatomic potentials (MLIPs) have been proposed to overcome the trade-off that has existed in physics-based computational models for many decades. MLIPs often achieve computational efficiency similar to classical FFs but with QM-level accuracy^1–16^. Among the many different types of MLIPs that have been proposed, neural network (NN)-based MLIPs are especially capable of describing a broad range of chemical systems without additional specialization and, thereby, represent a top candidate for developing a truly general MLIP. For example, ANAKIN-ME (or ANI) is a NN-based MLIP that has been trained to large and chemically diverse datasets of organic molecules containing the elements C, H, N, O, S, F and Cl (refs. ^17,18^). While previous ANI MLIPs proved to be extremely accurate for near-equilibrium conformations of organic molecules in vacuo, these potentials do not address the challenges of modelling condensed-phases (that is, periodic systems of liquids, supercritical fluids or solids) and reactive chemistry.

Several MLIPs have been developed for studying both condensed-phase and gas-phase (or in vacuo) reactive chemistry of a specific system^19–25^. However, each of these studies required considerable domain and MLIP expertise and enormous computational resources to build a non-general reactive MLIP. For this reason, a highly general reactive MLIP would be transformational towards the usage and impact of MLIPs among non-experts. While recent endeavours have yielded groundbreaking results towards a general MLIP for approximately one third of the periodic table^26–28^, these studies do not directly target reactive chemistry. Targeted, model-aware sampling strategies for dataset generation of three-dimensional atomic positions are especially essential for modelling rare events, such as chemical reactions^17,29^.

Active learning (AL)^30^ is a class of model-aware algorithms designed to automatically sample, select and label new data with the goal of efficiently generating a diverse and relevant dataset to train a more robust ML model. AL aims to ameliorate human bias through automating the decision-making process for adding new data to a training dataset. Recently, AL has been applied to develop numerous MLIPs trained to datasets of atomic positions labelled with energies and atomic forces from expensive QM calculations^17,19,31–36^.

To develop a general reactive MLIP with AL, existing methodologies for selecting, labelling and training are relatively straightforward to apply (Methods). However, for sampling atomic positions, adequately exploring reactive chemical space in an automated fashion is extremely challenging^37^ because it requires the exploration of chemical variance of molecular species in tandem with structural variance associated with non-equilibrium thermodynamic processes. The traditional approach of fitting reactive FFs^38–42^ to a limited dataset of pre-determined gas-phase reaction pathways based on chemical intuition is insufficient for developing a general reactive MLIP, as the resulting MLIP would be biased to only perform well on the assumed reaction network. Similarly, while recent work (performed simultaneous and independent to this study) presented an automated approach to sample transition states and minimum-energy-path structures for gas-phase (or in vacuo) reactions of C, H, N and O molecules^43^, this sampling procedure is unlikely to result in an MLIP that is robust for condensed-phase high-temperature reactive MD simulations. By contrast, training an MLIP directly to condensed-phase QM reactive data would ensure that the potential is reliable for the density ranges typically used in reactive MD simulations.

Wang and co-workers developed an elegant approach for the MD-based exploration of reaction pathways in the condensed phase, using QM methods, referred to as the ab initio nanoreactor (NR)^44,45^. The NR was designed to model high-velocity molecular collisions of small molecules by using a fictitious biasing force to promote chemical reactions and the formation of new molecules, thus automatically exploring reaction pathways between arbitrary reactants and products. The ab initio NR was successfully able to predict graphene ring formation from pure acetylene as well as reaction pathways to form glycine, one of the building blocks of life, from small early earth molecules.

**Fig. 1:**
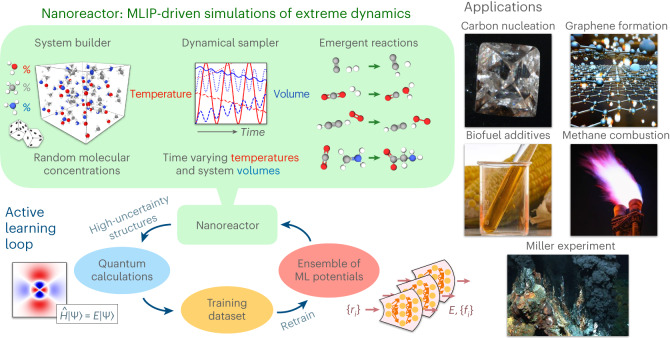
Summary of the nanoreactor active learning workflow and specific applications considered. The AL loop is an automated, iterative and efficient approach to develop a MLIP. AL generates a training dataset consisting of quantum calculations for only the high-uncertainty structures, as identified based on an ensemble of MLIPs. Structures relevant to condensed-phase reactive chemistry are sampled using NR simulations. The initial system is built by random configurations of small molecules consisting of the elements C, H, N, and O. Dynamic simulations are performed using the current MLIP with extreme fluctuations in temperature and volume to induce chemical reactions. To test the generality of the resulting model, the final MLIP is then applied to several case studies that were not directly targeted during training.

Although Wang et al. clearly demonstrated the promise of the ab initio NR to discover reactive chemistry, QM-driven MD sampling is extremely computationally intensive for generating a large training dataset within AL. In this Article, inspired by the work of Wang et al., we design an MLIP-driven NR sampling procedure that targets arbitrary reactive chemical processes and compositions of C, H, N and O elements, including near pure elemental systems and mixtures. Combined with the ANI model architecture and applying AL at scale, we aim to produce a robust and general reactive MLIP. Figure 1 shows a summary of the NR–AL workflow and the specific applications investigated in this work with the final model, referred to as ANI-1xnr.

To evaluate the reliability of ANI-1xnr in practical research scenarios, we conduct several condensed-phase reactive chemistry simulations inspired by other literature with the ANI-1xnr potential, namely carbon solid-phase nucleation^46–48^, graphene ring formation from acetylene with varying O_2_ concentrations^44,49^, biodiesel ignition with different fuel additives^50^, methane combustion^24^ and the spontaneous formation of glycine from early earth molecules^44,51,52^. Across this wide range of applications, we show that ANI-1xnr provides results that are consistent with chemical intuition, experimental data, QM calculations (density-functional theory (DFT), Hartree–Fock and density-functional tight-binding (DFTB)) and classical reactive MD simulations (reactive force field (ReaxFF) and an application-specific MLIP) all without retraining.

This study demonstrates the capability of automated chemical exploration workflows to build a general-purpose reactive potential, resulting in ANI-1xnr, a fast, accurate and general potential capable of simulating a wide range of real-world reactive systems containing C, H, N and O elements.

## Results

### Nanoreactor active learning

**Fig. 2:**
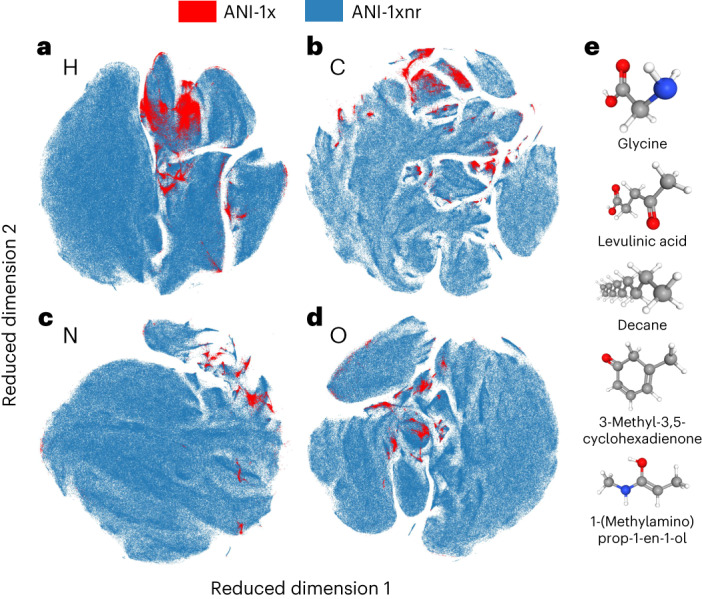
Analysis of the dataset generated in this work (ANI-1xnr) with nanoreactor active learning. **a**–**d**, A comparison between the ANI-1xnr dataset (blue points) and a non-reactive, near-equilibrium, molecule in vacuo, AL dataset from the literature (ANI-1x; red points). Two-dimensional visualizations of the local atomic environments for the elements H (**a**), C (**b**), N (**c**) and O (**d**). The ANI-1xnr dataset not only encompasses the vast majority of the regions sampled in the ANI-1x dataset, but it also interpolates between these regions and even extends these regions substantially. For visual clarity and to manage memory loads, only a random subset of the ANI-1x dataset and ANI-1xnr dataset are depicted in **a**–**d**. **e**, Five examples of the over 1,000 unique molecules that formed during AL. Reaction pathways to form these molecules must, therefore, be present in the ANI-1xnr dataset.

Before assessing the performance of the ANI-1xnr model on the different case studies, we evaluated both the diversity and the completeness of the ANI-1xnr dataset. Figure 2 provides a two-dimensional visualization of a high-dimensional dataset by clustering together similar local atomic environments for the elements H (Fig. 2a), C (Fig. 2b), N (Fig. 2c) and O (Fig. 2d). Figure 2a–d compare the ANI-1xnr dataset and a non-reactive, near-equilibrium, molecule in vacuo, AL dataset (ANI-1x). Clearly, the ANI-1xnr dataset not only effectively encompasses the entire ANI-1x dataset but it also extends substantially beyond the local atomic environment space covered by ANI-1x. More importantly, the ANI-1xnr dataset provides pathways between many of the clusters in the ANI-1x dataset. These pathways probably correspond to reactions in a low-dimensional representation. Furthermore, Fig. 2e provides select examples of the over 1,000 unique molecules (consisting of ten or fewer CNO atoms; Methods) that are identified in the ANI-1xnr training dataset. Since the NR sampling simulations were initialized with only small molecules (consisting of two or fewer CNO atoms; Methods), the NR–AL procedure automatically discovered hundreds, if not thousands, of reaction pathways leading to these distinct molecular structures.

### Carbon solid-phase nucleation

Accurate simulation of amorphous carbon systems has long been one of the top interests among chemists and materials scientists, as some distinct materials (for example, graphene, diamond and carbon nanotubes) form from amorphous carbon under different conditions. Understanding this behaviour would assist in the development of functional materials by controlling the solid-phase nucleation process. Many reactive FFs have been employed to simulate amorphous carbon in MD^48,53,54^. With the widespread use of ML methods, researchers recently developed application-specific MLIPs to investigate amorphous carbon systems^46,55^. These application-specific MLIPs proved accurate at predicting pure carbon fragments and mechanical properties of the bulk system. Despite these achievements, MLIPs trained on application-specific datasets would have very poor generality to new chemistry as the model has only been fit to a limited number of structures and reactions. On the other hand, the NR–AL approach presented in this work does not sample any specific form of carbon explicitly. We rely on the NR sampler and AL algorithm to automatically select physically relevant and unbiased configurations of carbon atoms. To validate ANI-1xnr in carbon solid-phase nucleation simulations under different conditions, we perform simulations at high (3.52 g cc^−1^), medium (2.25 g cc^−1^) and low (0.50 g cc^−1^) densities.

**Fig. 3:**
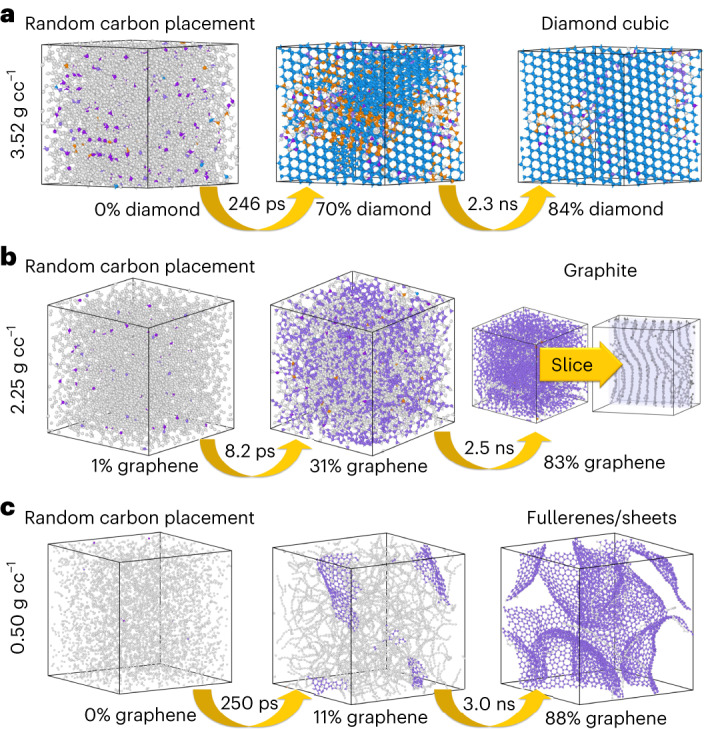
Carbon solid-phase nucleation simulation results for ANI-1xnr. **a**–**c**, Specific densities at 3.52 g cc^−1^ (**a**), 2.25 g cc^−1^ (**b**) and 0.5 g cc^−1^ (**c**). Simulations are initiated with random carbon positions. The final structures agree with the expected phases of carbon for each density. Specifically, **a** produces diamond cubic crystal, **b** produces graphite-like graphene sheets and **c** produces fullerene-like graphene sheets.

Figure 3 summarizes the product of each simulation. For each of the high- (Fig. 3a), medium- (Fig. 3b) and low-density (Fig. 3c) carbon simulations, ANI-1xnr produces the expected structure of carbon for the respective density^46–48^. Specifically, for the system with the highest density (3.52 g cc^−1^), diamond, graphene and hexagonal diamond phase coexist after 246 ps, where 70% of carbon atoms in the simulation box forms diamond cubic crystal structure. After another 2.3 ns, the high-density system contains 86% of carbon atoms in the diamond cubic crystal structure, with very few graphene and hexagonal diamond sites. In the medium-density (2.25 g cc^−1^) system, 31% of atoms rapidly form graphene after 8.2 ps, and the system contains 83% graphene after another 2.3 ns. Graphene sheets tend to form a stacked and more ordered graphite-like structure, which is observed for the system slice in Fig. 3b). The low-density (0.5 g cc^−1^) system forms carbon atom chains after 250 ps, with 11% of atoms forming graphene sheets. After another 3 ns, the system contains 88% of atoms formed in graphene sheets. However, the graphene sheets in this low-density case are more disordered and appear to form fullerene-like closed or partially closed meshes. Extended Data Table 1 provides an analysis of the ANI-1xnr crystal lattice constants for diamond and graphite.

### Effect of oxygen on graphene ring formation

Wang et al.^44^ applied the original ab initio NR method to observe ring formation (that is, the early stages of graphene formation) from a pure acetylene (C_2_H_2_) system. Subsequently, Lei et al.^49^ presented DFTB NR simulations of acetylene in the presence of different amounts of oxygen, where O_2_/C_2_H_2_ = 0, 0.1, …, 1 is the ratio of added O_2_ while the number of C_2_H_2_ molecules is fixed to 40. Graphene formation is the dominant process for pure C_2_H_2_, as the generation of free radicals enables the rapid growth of hydrocarbon rings. By contrast, the addition of O_2_ to the system deters or, at high enough O_2_/C_2_H_2_ ratios, completely eliminates ring formation^49^. Similar to the work of Lei et al.^49^, we perform reactive simulations with varying ratios of C_2_H_2_ and O_2_.

**Fig. 4:**
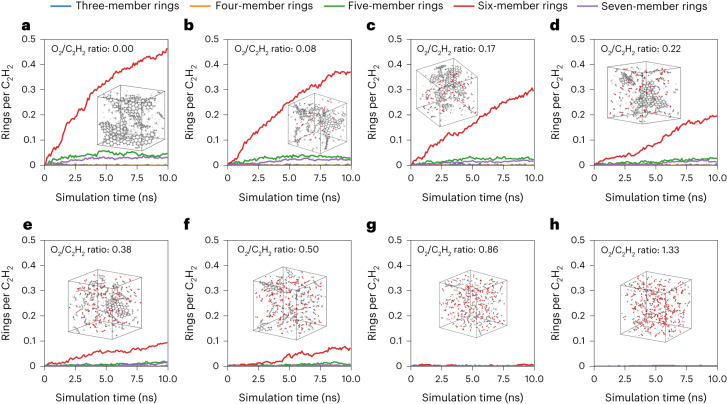
Effect of oxygen on graphene ring formation simulation results for ANI-1xnr. **a**–**h**, A comparison of three-, four-, five-, six- and seven-membered ring formation for different ratios of O_2_/C_2_H_2_: 0.00 (**a**), 0.08 (**b**), 0.17 (**c**), 0.22 (**d**), 0.38 (**e**), 0.50 (**f**), 0.86 (**g**) and 1.33 (**h**). ANI-1xnr predicts six-membered ring formation for O_2_/C_2_H_2_ ratios less than 0.50, in closer agreement with experimental data than the DFTB simulation results of Lei et al.^49^ In comparison with these literature DFTB simulations, the computational efficiency of ANI-1xnr enables considerably longer simulation times and larger systems. Specifically, while Lei et al. performed simulations of 0.5 ns with between 160 and 270 atoms (depending on the O_2_/C_2_H_2_ ratio), we simulate 1,000 atoms for 10 ns (Methods). Source data

**Extended Data Fig. 1:**
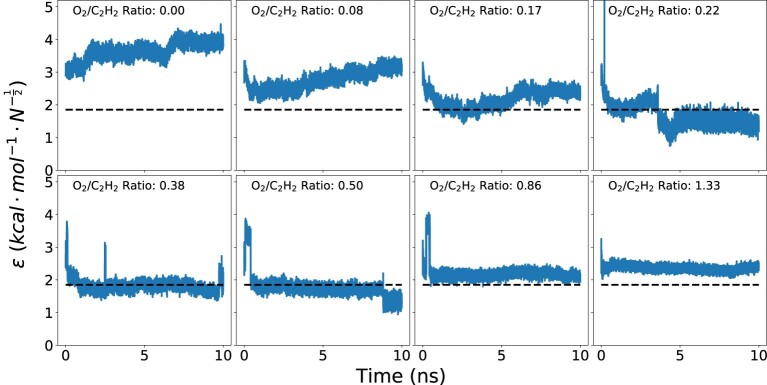
ANI-1xnr uncertainty for all eight O_2_/C_2_H_2_ ratios in graphene ring formation simulations. Uncertainty (*ε*) is computed as ensemble standard deviation in energy normalized by the square-root of number atoms. Dashed black line corresponds to AL energy threshold of 1.85 ${\rm{kcal}}\,\,{\rm{mol}}^{-1}\,\,{N}^{-\frac{1}{2}}$. The MLIP uncertainty is fairly constant and approximately equal to the AL energy threshold through nearly the entire simulation (with only a few snapshots as exceptions). The relatively low and constant uncertainty confirms that each system is well-modeled by our MLIP. It is also interesting that the MLIP uncertainty decreases with increasing O_2_/C_2_H_2_ ratio, suggesting that ANI-1xnr is most confident under a higher O_2_/C_2_H_2_ ratio. The reason for such a tendency is that more oxidation reactions happen in the system with a larger O_2_/C_2_H_2_ ratio, which is a common reaction in our training dataset, although typically involving species other than acetylene. By contrast, the system with a smaller O_2_/C_2_H_2_ ratio has more ring formation, large carbon sheet formation and even phase change process, which are less common in the training set. Although C_2_H_2_ and O_2_ are common species in the ANI-1xnr dataset (see Methods for details), the fact that the uncertainties remain slightly larger than those for any structure in the entire ANI-1xnr training set demonstrates that these specific systems were not studied directly in the NR AL sampling. Therefore, this case study serves as an assessment of the generality of the ANI-1xnr potential.

Figure 4 shows the amount of three-, four-, five-, six- and seven-membered rings formed with respect to simulation time for eight different O_2_/C_2_H_2_ ratios. Increasing the oxygen ratio decreases the number of rings formed, which is in good agreement with the simulations from Lei et al. and experimental literature^56^. Furthermore, although the branching ratios (that is, the relative production of different ring sizes) are not completely converged for all systems, the branching ratios are clearly in qualitative agreement with Lei et al. Specifically, six-membered rings are the predominant product, followed by five-membered and seven-membered rings at noticeably lower, but nearly equal, branching ratios. However, in contrast with Lei et al., six-membered rings form even for an O_2_/C_2_H_2_ ratio of 0.5. In comparison, the simulations of Lei et al. predict ring formation for O_2_/C_2_H_2_ ratio up to 0.2, but negligible ring formation for an O_2_/C_2_H_2_ ratio of 0.4. The ANI-1xnr results are in much closer agreement with experimental data, which report graphene formation for O_2_/C_2_H_2_ ratios between 0.4 and 0.8 (no experimental measurements were reported outside of this range). A clear explanation for the improved agreement between ANI-1xnr and experiment is the longer simulation timescales and the larger system sizes achievable by ANI-1xnr compared with DFTB (Methods). Specifically, for an O_2_/C_2_H_2_ ratio of 0.5, six-membered rings only begin to form after 1 ns with ANI-1xnr. Considering that the DFTB simulations of Lei et al. ran for only 0.5 ns, our results suggest that six-membered rings could form under higher oxygen ratio conditions using DFTB at longer timescales. Although it is possible that even longer MD simulations could result in ring formation at even higher O_2_/C_2_H_2_ ratios, this case study demonstrate the value in the lower computational costs of ANI-1xnr compared with traditional methods, such as DFTB, to discover interesting phenomena that can only be observed during long timescale simulations. Further validation of the ANI-1xnr simulation results are provided in Extended Data Fig. 1.

### Comparison of biofuel additives

To promote combustion processes of liquid fuel, fuel additives are utilized as detergents, oxygenates, emission depressors, corrosion inhibitors, dyes and to increase the octane number. Chen et al.^50^ performed high-temperature high-pressure MD simulations with ReaxFF^40,42^ to predict the mechanisms and kinetics of several fuel additives, including ethanol, 2-butanol and methyl *tert*-butyl ether (MTBE). According to their results, 2-butanol was the best fuel additive at enhancing ignition while MTBE demonstrated similar ignition enhancement to 2-butanol. By contrast, ethanol was the worst fuel additive, having a negligible effect on the O_2_ consumption rate and ignition delay time (IDT) compared with the clean biofuel.

**Fig. 5:**
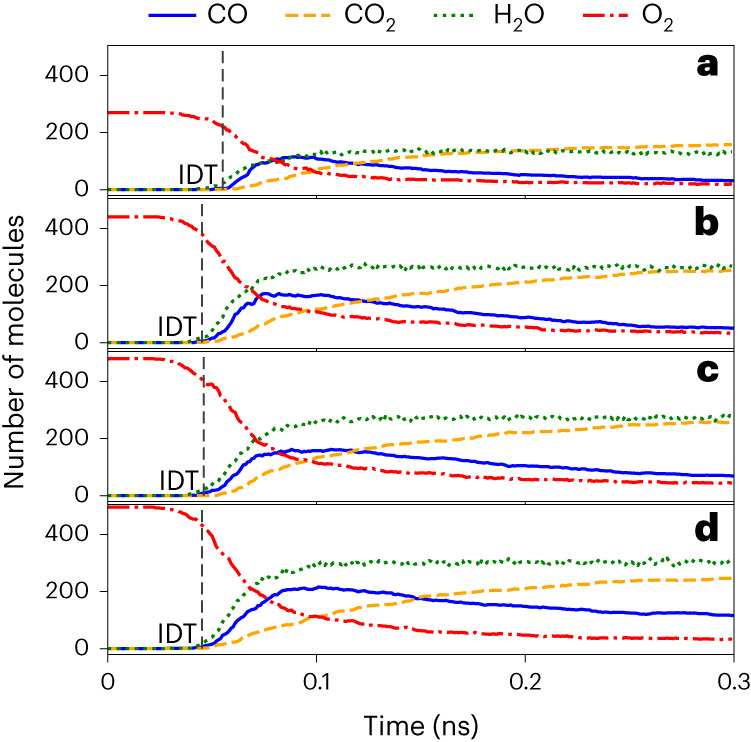
Biofuel additive simulation results for ANI-1xnr. **a**–**d**, Tracking plots of O_2_ and major products (CO, CO_2_ and H_2_O) for the following biofuel simulations: biofuel+O_2_ (**a**), biofuel + O_2_ with ethanol additive (**b**), biofuel + O_2_ with 2-butanol additive (**c**) and biofuel + O_2_ with MTBE additive (**d**). IDT is defined as the average time that at least five molecules of CO, CO_2_ and H_2_O are produced (Supplementary Fig. 1). IDT is significantly decreased for each additive in comparison with the clean biofuel. For tracking plots including the entire 2 ns simulation, see Extended Data Fig. 2. Source data

**Extended Data Fig. 3:**
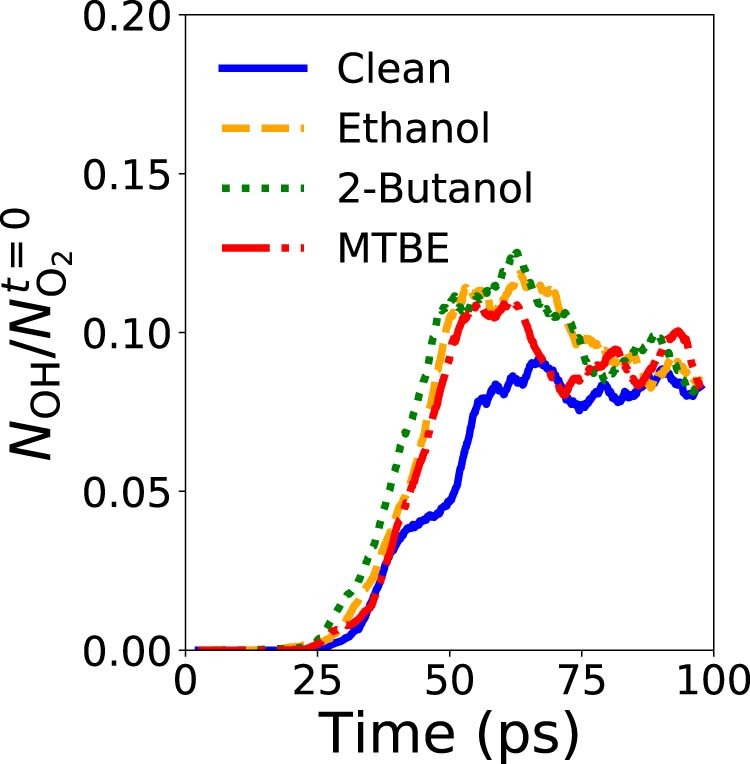
Comparison of normalized production rate of OH for biofuel additive simulations. Number of OH species (*N*_OH_) is normalized by the initial number of O_2_ molecules $({N}_{{{{{\rm{O}}}}}_{2}}^{t = 0})$. In comparison to the pure biofuel, all three systems with additives have a higher and earlier peak in OH radical, when normalized by the initial amount of O_2_. The enhancement in OH production for ethanol is intuitive since ethanol contains a hydroxyl group with a similar bond dissociation energy to 2-butanol. Considering the important role that the OH radical plays in ignition and combustion chemistry, the accelerated rate of OH production is consistent with a lower IDT for all three additive systems. Thus, although the ANI-1xnr results for ethanol are in conflict with ReaxFF, the enhancement in OH production provides understanding as to how ethanol accelerates the ignition process, similar to 2-butanol and MTBE.

To validate the reliability of ANI-1xnr for simulating biodiesel and to investigate the reported ignition enhancement of fuel additives, we reproduced four systems simulated by Chen et al.^50^, namely, clean biodiesel, biodiesel with ethanol as additive, biodiesel with 2-butanol as additive and biodiesel with MTBE as additive. Figure 5 shows that the main products (CO, CO_2_ and H_2_O) are produced in very similar quantities to the ReaxFF simulations of Chen et al. Despite a quantitative difference between ANI-1xnr and ReaxFF IDTs (Extended Data Table 2), the additive effect on ignition delay for ANI-1xnr agrees qualitatively with ReaxFF, namely, all three additives cause product formation to occur at earlier times compared with clean biodiesel. Furthermore, ANI-1xnr predicts that 2-butanol and MTBE both result in the enhancement of O_2_ consumption, similar to ReaxFF (Extended Data Table 2). The primary qualitative discrepancy with ReaxFF is that ANI-1xnr predicts that ethanol also enhances O_2_ consumption. However, experimental work demonstrates that ethanol can actually accelerate fuel ignition at relatively high pressures, in agreement with our simulation results^57^. Extended Data Fig. 3 provides further justification for the ANI-1xnr ethanol simulation results.

### Methane combustion

**Fig. 6:**
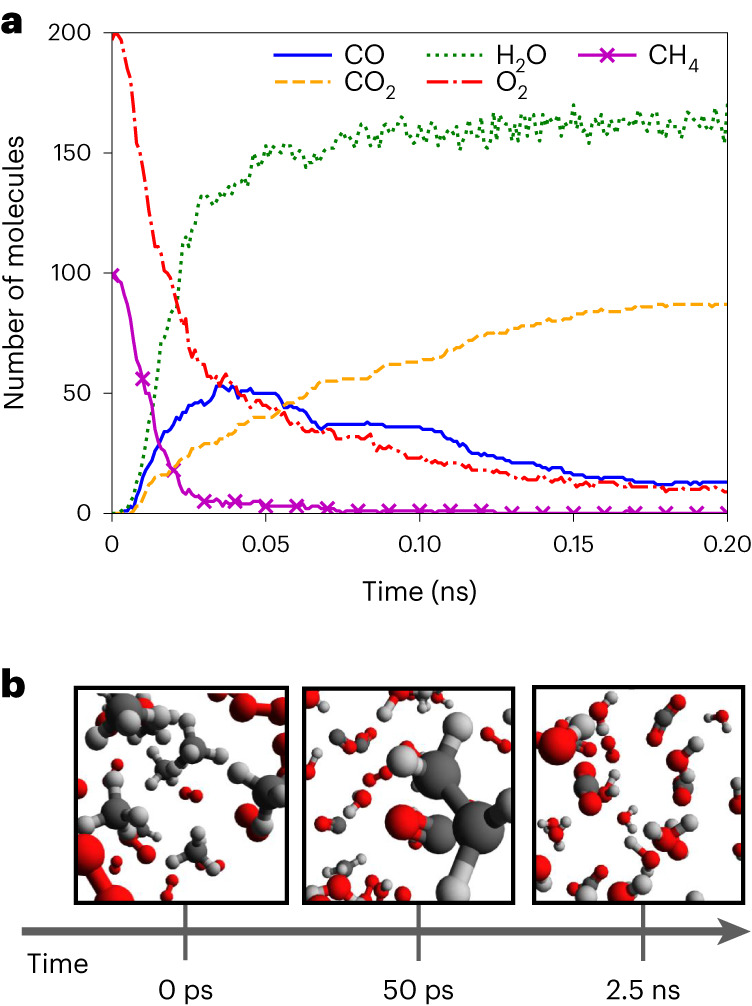
Methane combustion simulation results for ANI-1xnr. **a**, A tracking plot of O_2_, CH_4_ and major products (CO, CO_2_ and H_2_O). The tracking plot for the full simulation is provided as Supplementary Fig. 3. **b**, Snapshots of initial reactants, intermediate species and final products. Both the tracking plot and the snapshots confirm that ANI-1xnr predicts qualitatively reasonable reactive chemistry for this system. However, ANI-1xnr is markedly more reactive than the application-specific MLIP in the literature^24^. Source data

**Extended Data Fig. 4:**
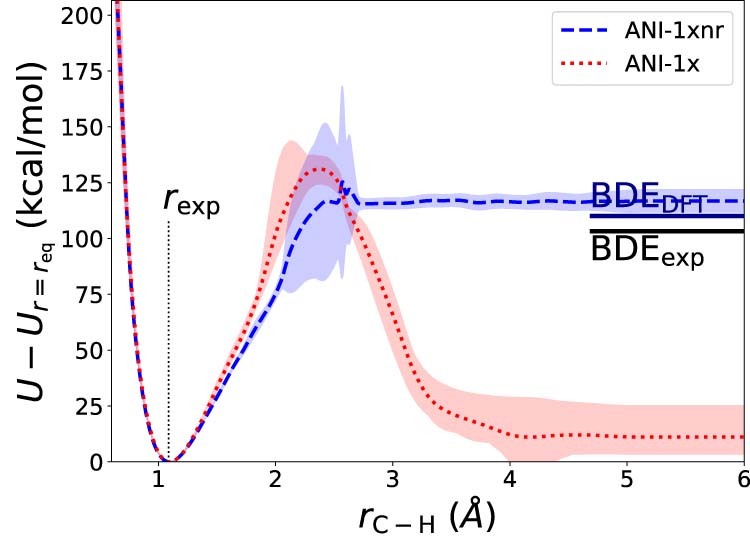
Bond dissociation diagram for C-H bond in methane. Data are presented as mean (center line) ± ensemble standard deviation (shaded region). Comparison between ANI-1xnr and ANI-1x with the DFT and experimental bond dissociation energies (BDE). The C-H bond dissociation energy from ANI-1xnr (116.6 kcal mol^−1^) is similar to that from our reference DFT (110.0 kcal mol^−1^), the reference DFT of Zeng et al. (115.0 kcal mol^−1^), and experiment (103.3 kcal mol^−1^). Although an *in vacuo* energy barrier may not correspond to a condensed-phase reaction rate, this analysis demonstrates that the accelerated consumption of CH_4_ is not due to a vastly under predicted C-H bond dissociation energy by ANI-1xnr or our reference DFT. However, the relatively large uncertainties in ANI-1xnr along the bond dissociation path suggest that ANI-1xnr should not be utilized for studying low-density gas-phase (or *in vacuo*) bond-breaking reactions. Although ANI-1xnr exhibits markedly greater uncertainty between 2-3 Å, the MLIP remains smooth and the forces are continuous. The poor performance in this high-uncertainty region is possibly due, in part, to the fact that the spin multiplicity of the minimum-energy electronic state changes from a singlet to a triplet in the gas-phase. Whereas, ANI-1xnr does not explicitly learn the relationship between energy and spin multiplicity and was trained on condensed-phase singlet data, where the notion of spin is much less pronounced. Although this may seem surprising that ANI-1xnr performs worse for *in vacuo* calculations where the physics are definitively simpler than for the condensed-phase, it is important to recall that the performance of MLIPs is highly dependent on having relevant training data. Specifically, since ANI-1xnr was trained solely to condensed-phase NR simulations (with densities between 0.5 and 2.5 g cc^-1^), we do not recommend using ANI-1xnr for *in vacuo* calculations.

**Extended Data Fig. 5:**
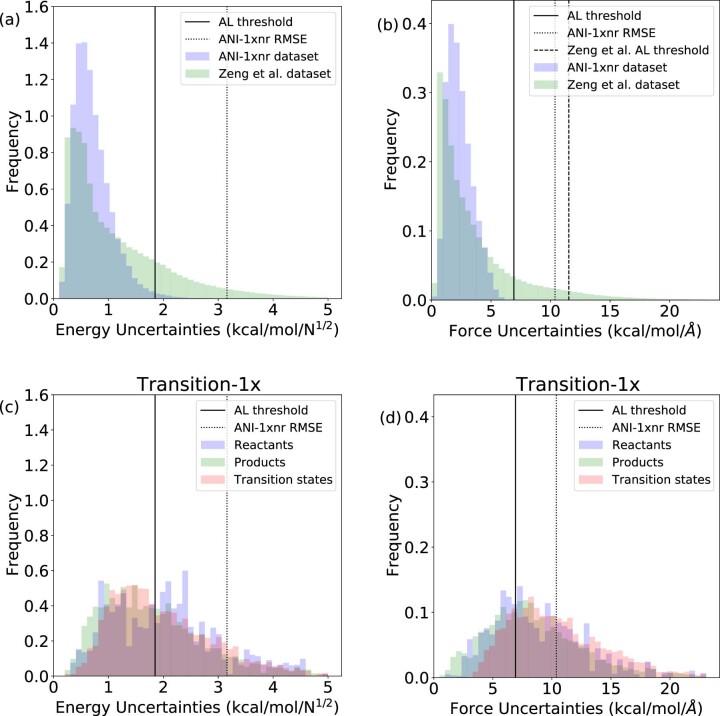
Uncertainty analysis of ANI-1xnr on reactive literature datasets. Distribution of ANI-1xnr ensemble uncertainties in (a/c) energy and (b/d) force for the (a/b) ANI-1xnr dataset, (a/b) Zeng et al. dataset for methane, and (c/d) Transition-1x reactive dataset. Energy uncertainty is the ensemble standard deviation for energy normalized by the square-root of number atoms. Force uncertainty is the ensemble standard deviation for force averaged over all atoms and Cartesian coordinates. The energy and force uncertainties in (a/b) for the Zeng et al. dataset are smaller than the AL thresholds of 1.85 ${\rm{kcal}}\,\,{\rm{mol}}^{-1}\,\,{N}^{-\frac{1}{2}}$ and 6.92 kcal mol^−^^1^ A^−^^1^, respectively, for approximately 77% of the ≈ 567000 structures. Note that the AL selection criterion of Zeng et al. was based solely on the force uncertainty. The ANI-1xnr force uncertainty is larger than the Zeng et al. AL threshold of 11.53 kcal mol^−^^1^ A^−^^1^ for only 5% of the structures in the Zeng et al. dataset, suggesting that ANI-1xnr has a similar confidence on this dataset as the application-specific MLIP of Zeng et al. Note that with such a high AL threshold, it is quite likely that there are some non-physical structures in the Zeng et al. dataset. Furthermore, the Zeng et al. dataset also consists of structures sampled with ReaxFF without curation. It is also important to recall that the Zeng et al. training dataset consists of small clusters of molecules extracted from a condensed-phase MD simulation. Therefore, atoms near the center of the cluster are in condensed-phase environments while atoms on the border of the cluster are effectively in a gas-phase environment. By contrast, the unilateral high uncertainties for the Transition-1x dataset in (c/d) demonstrate that ANI-1xnr is not intended for *in vacuo* reactive chemistry. Note that the distribution of Transition-1x uncertainties is nearly the same for reactants, transition states, and products, demonstrating that the issue is the vacuum environment rather than unsampled transition states.

Emerging research has shown the success of application-specific MLIPs on systems such as radical reactions in hydrocarbon combustion and well-known gas-phase mechanisms^58,59^. Zeng et al.^24^ trained an NN-based potential to a dataset of QM-calculated fragment clusters sampled from a ReaxFF simulation of the combustion process of a mixture of CH_4_ and O_2_. They showed that their application-specific MLIP could then simulate the combustion process of methane with a reasonable mechanism. Though our ANI-1xnr potential was trained for a more general purpose, we compare the performance of our MLIP with the application-specific MLIP of Zeng et al. for methane combustion under high temperatures and pressures. Specifically, we reproduce their MD simulation of methane combustion under the same conditions with the ANI-1xnr potential. Figure 6a shows that the ANI-1xnr potential produces very similar major products and species profiles to those of Zeng et al. However, by comparison with the CH_4_ and O_2_ consumption rates of Zeng et al., ANI-1xnr predicts an overall reaction rate that is approximately a factor of 40 times faster. Specifically, while their system required 0.5 ns of simulation time to consume half of the initial CH_4_, our system required only 0.012 ns. Similar to the biofuel case, the difference in the overall reaction rate is probably due to the difference in the reference DFT reaction energy barriers (Methods). Extended Data Figs. 4 and 5 provide further explanation as to the potential cause of this discrepancy.

Due to the extreme simulation conditions, no experimental reference data are available for comparison. However, the similar trend for species concentration with respect to time in comparison with the work of Zeng et al. indicates that our general-purpose MLIP was able to learn the relevant physics and mechanisms as well as the application-specific MLIP of Zeng et al. Also, the CH_4_ and O_2_ consumption curves for the ANI-1xnr model are much closer to exponential decay, which is more physically reasonable than the near-linear decay plots of Zeng et al.

### Miller experiment

In 1959, Stanley Miller designed a famous experiment to elucidate the origins of life on earth^51^. Miller applied an electric field to a gaseous system consisting of simple small-molecule species (for example, NH_3_, CO, H_2_O, H_2_ and CH_4_) and reported the formation of amino acids such as glycine (C_2_H_5_NO_2_). This revolutionary experiment led to the formation of the field of prebiotic chemistry, which aims to discover the reaction networks that produce molecules that are essential for the formation of life. In this spirit, computational studies have attempted to imitate the reaction conditions of the Miller experiment to predict the key reaction pathways that lead to the formation of glycine. Recently, Saitta and Saija performed relatively short (≈40 ps) near-ambient temperature (400 K) condensed-phase (≈1 g cc^−1^) DFT-MD simulations, wherein an electric field is applied directly to ‘spark’ chemical reactions^52^. As our MLIP does not contain the necessary electronic information to apply an electric field, we instead encourage reactions to occur on picosecond timescales by performing high-temperature high-density MD simulations, similar to the Miller NR simulation of Wang et al.^44^ Due to the low computational cost of our MLIP, we are able to run our Miller experiment simulation considerably longer (≈4 ns) than the ab initio NR simulations of Wang et al. (≈1 ns) with the same system size of 228 atoms but with periodic boundary conditions. For this reason, we use a constant condensed-phase density (with corresponding pressures around 1 GPa) rather than applying an artificial piston to periodically compress the non-periodic gas-phase system to around 10 GPa, as was the approach employed by Wang et al.

**Fig. 7:**
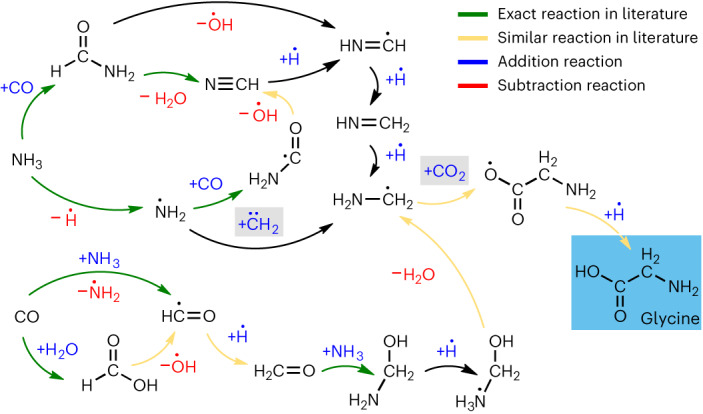
Miller experiment simulation results for ANI-1xnr. The reaction pathways discovered by ANI-1xnr in a Miller experiment simulation for the formation of glycine from small-molecule species (for example, NH_3_, CO, H_2_O, H_2_ and CH_4_). The green arrows denote reactions previously identified by Wang et al. or Saitta and Saija. The orange arrows denote reactions that have a similar reaction in Wang et al. or Saitta and Saija. The majority of reactions have been previously reported in the literature, confirming the validity of the ANI-1xnr mechanism. Three-dimensional snapshots extracted from the MD simulation trajectory are reported in Extended Data Fig. 6, further confirming that the reaction pathways are physically meaningful. Note that +H does not necessarily signify a free hydrogen atom, +H is short-hand for a proton donor, for example, NH_4_, NH_3_, CHO, CHNO, H_3_O or H_2_O. Likewise, −H does not necessarily signify dissociation of a hydrogen atom. −H is short-hand for a proton acceptor, for example, NH_2_, CO, CNO, H_2_O or OH. The boxes encapsulate the key intermediates, carbon dioxide (CO_2_) and methylene (CH_2_). The novel pathways to form these key intermediates are reported in Extended Data Fig. 7. The depiction of bond orders and radical species is based simply on chemical intuition, since ANI-1xnr does not provide explicit bonding, orbital or electronic information (for an alternative interpretation of this mechanism involving ionic species, see Extended Data Fig. 8).

Figure 7 shows the ANI-1xnr reaction mechanism to form glycine starting from the initial reactants. During our Miller simulation, glycine is formed three times and persists for approximately 225 fs, 375 fs and 913 fs. Dissociation of glycine in less than 1 ps is expected, considering the relatively high temperature of this simulation. The final step to form glycine is hydrogen addition to C_2_H_4_NO_2_, similar to the mechanism of Saitta and Saija. However, hydrogen addition occurs at an oxygen atom in our mechanism, rather than at the *α*-carbon as in the Saitta and Saija mechanism. In one instance, our Miller simulation produced the same C_2_H_4_NO_2_ isomer as reported by Saitta and Saija. By contrast to the Saitta and Saija mechanism, this C_2_H_4_NO_2_ isomer dissociated in our simulation rather than forming glycine. The key precursor to C_2_H_4_NO_2_ is CH_4_N, which is formed through several pathways. The pathway to form CH_4_N that proceeds through the CH_2_O intermediate is very similar to the mechanism reported by Wang et al.^44^ The mechanisms to form the intermediates formaldehyde (CH_2_O) and hydrogen cyanide (CHN) from the initial reactants CO, NH_3_ and H_2_O were nearly identical to those reported by Wang et al.^44^ and Saitta and Saija^52^. Overall, there are several similarities between our mechanism and those of Wang et al. and Saitta and Saija.

## Conclusions

Here, we introduced a sampling procedure, dataset and MLIP (ANI-1nxr) based on the NR for organic condensed-phase MDs, including reactions. The NR-based AL process builds a reactive dataset spanning elemental compositions of C, H, N and O under a wide range of conditions starting from nine small seed molecules. The NR–AL procedure provided data with unprecedented chemical environment diversity and relevance compared with prior non-reactive AL, and uncovers more than 1,000 unique molecules in total, under condensed-phase reactive atomistic configurations. Each unique molecular species formed by MDs simulation in our NR sampler was the result of one or more reaction pathways that did not need to be known or specified before runtime.

We validated the generality of the ANI-1xnr potential on five real-world condensed-phase reactive case studies: carbon solid-phase nucleation, effect of oxygen on graphene ring formation from acetylene, ignition of biodiesel with various fuel additives, combustion of methane and the spontaneous formation of glycine in early earth conditions, all without retraining. In carbon solid-phase nucleation and graphene ring formation studies, we show that ANI-1xnr reproduces the experiment well. In other cases, in extreme simulation conditions where an experiment is not available for comparison, ANI-1xnr produces results that are generally consistent with traditional modelling approaches, such as DFT, DFTB, ReaxFF and even an application-specific MLIP. The effectiveness of the NR–AL approach demonstrates the power of coupling and automating the system exploration, data generation and model training processes to produce a robust MLIP.

Although the ANI-1xnr potential is already a broadly applicable tool for studying condensed-phase reactive chemistry, we envision continuous improvement of this MLIP. Future work could augment the condensed-phase ANI-1xnr dataset with low-density or in vacuo reactive data, for example, by sampling pathways for pre-determined reactions^37,43^ or for reactions identified in the NR simulations^44^. Future work could also extend the dataset to additional elements^18^. As the current dataset was computed using an affordable plane-wave DFT method, future work could also investigate the prospect for higher-accuracy QM methods (for example, double-hybrid DFT or post-Hartree–Fock) to obtain improved reaction barriers. In addition to simple retraining, any of these improvements could use more advanced ML training paradigms, such as transfer learning^60^, meta-learning^61^ and lifelong learning^62^. Concerning the model form, the ANI-1xnr potential is fully local, meaning long-range effects, such as London dispersion and Coulombic interactions, are not described explicitly beyond the model cutoff radius (Methods). Certain applications may require more direct treatment of long-range effects. Future work could investigate incorporating recent developments, such as explicit long-range terms^63^, charge equilibration schemes^64^ or graph NN models^5,6,13–16^ that can implicitly account for long-range interactions. A recent advancement in ML for natural language processing is the concept of foundational models, that is, large, general models usually trained with unlabelled data that can be specialized to specific tasks quickly with very small amounts of data^65^. As ANI-1xnr is trained to a large, general dataset, a clear future direction is to evaluate whether it can act as a foundational model for application-specific MLIPs when greater accuracy is required.

We are providing the ANI-1xnr dataset for future research. We are also providing the resulting ANI-1xnr potential to the community. We advise potential users to exercise strong caution if applying ANI-1xnr outside of the training domain (CHNO condensed-phase reactive chemistry). Nonetheless, considering that ANI-1xnr was developed independently of the five case study systems, the generality of ANI-1xnr is truly remarkable.

## Methods

### Model description and training details

The ANI-1xnr model was trained similarly to ANI models within other contexts^18^, including materials science^32^ and chemistry^66^. We use the ANI descriptors^67^, which is a modified form of the Behler and Parinello NN descriptors^12^. ANI-1xnr uses a local cutoff of 5.2 Å for the radial descriptors and 3.5 Å for the angular descriptors. The model is trained for the elements C, H, N and O, each of which has its own specialized NN-based potential. The NN architecture for each element and symmetry functions are reported as Supplementary Tables 2 and 3, respectively. Similar to previous ANI models, ANI-1xnr predicts energies based solely upon the atomic positions and element types. Therefore, unlike more complex MLIPs, for example, SpookyNet^16^ and AIMNet^68^, the ANI-1xnr-predicted energies do not explicitly depend on the charge or spin multiplicity. Thus, similar to ReaxFF, ANI-1xnr predicts only the ground-state energy, regardless of whether the lowest-energy electronic state corresponds to a radical or an ion.

#### Training

The ANI-1xnr model was trained using both energy and force terms in the loss function as described in previous work^69^. During training, we employ early stopping to prevent overfitting with learning rate annealing to ensure a high-fidelity fit. The model training is considered converged when the learning rate drops below 1.0 × 10^−5^. Model performance against the held-out test dataset is presented in Supplementary Tables 4 and 5 and Supplementary Figs. 5–8. Note that, similar to previous MLIP studies^27,28^, we report the per-atom energy errors. This is because energy is an extensive property and the ANI-1xnr dataset consists of systems spanning almost two orders of magnitude in the number of atoms. Therefore, the unnormalized energy spans an enormous range and, thus, the corresponding unnormalized energy error is skewed by larger systems.

Although the ANI-1xnr root mean squared errors are approximately an order of magnitude larger than an MLIP trained to near-equilibrium data (for example, ANI-1x). This is not surprising since the ANI-1xnr dataset is considerably more challenging to train due to the wider range of atomic environments and system energies present in a reactive dataset. While errors between MLIPs trained on different datasets are not precisely comparable, we note that the ANI-1xnr mean absolute errors are of similar magnitude to those for TeaNet^27^, which was also trained on a very challenging dataset aimed at developing a universal potential. Furthermore, the ANI-1xnr dataset is much more general than most reactive datasets that are limited to a single system of interest, for example, the Zeng et al. reactive dataset for CH_4_ + O_2_. By comparison, the force root mean squared errors of ANI-1xnr are only about 30% higher than those of the MLIP trained to the Zeng et al. single-reactive-system dataset, despite the ANI-1xnr dataset covering a substantially wider range of reactive chemistry. A validation that ANI-1xnr conserves energy is shown in Supplementary Fig. 9.

### Training dataset generation

The ANI-1xnr training dataset was generated through an iterative AL process, where sampling of new atomic configurations is obtained with NR-inspired MD simulations. To bootstrap the AL process, periodic cells containing randomly placed and oriented small molecules with less than three non-H atoms and with a randomly selected composition of C, H, N and O are generated. Starting from this small initial bootstrap dataset, the AL algorithm is applied iteratively, yielding generations of datasets designed to improve upon their ancestors. Iterations of sampling, selecting, labelling and training are performed until the resulting MLIP is no longer improving. Training was described in the previous section. Details regarding sampling, selecting and labelling are provided in the following paragraphs.

#### Sampling

Atomic structures (that is, positions) are sampled by performing NR simulations with the current AL-generation MLIP. The MLIP-driven NR simulations are initialized with random compositions of small molecules, containing in the order of 100 atoms. Random oscillations of temperature and density (that is, simulation box volume) promote reactions and the formation of new products during the allotted simulation time (less than 1 ns).

#### Selecting

From all the atomic structures sampled along the NR trajectories, only high-uncertainty structures are selected for the ever-growing dataset, as these structures are deemed to be poorly described by the current AL-generation MLIP. Similar to previous ANI studies, we utilize a query-by-committee^70^ uncertainty metric, that is, the normalized ensemble standard deviation in energy and atomic forces^32,60^. To achieve a balance between exploration of chemical space and exploitation of the most important regions of the potential energy surface, the uncertainty thresholds vary between AL iterations, where the latter AL iterations generally have larger thresholds than earlier iterations. The final uncertainty threshold values for the normalized energy and forces were 1.85 ${{{\rm{kcal}}}}\; {{{{\rm{mol}}}}}^{-1}\; {{\mathrm{N}}}^{-\frac{1}{2}}$ and 6.92 kcal mol^−1^ Å^−1^, respectively.

#### Labelling

Each selected structure is then labelled with QM system energy and atomic forces. These QM calculations are computed with the open-source CP2K software^71^ using unrestricted Kohn–Sham DFT^72^, Becke, Lee, Yang and Parr (BLYP) functional^73,74^, triple-zeta valence basis set with two sets of polarisation functions (TZV2P)^75^, Goedecker, Teter and Hutter (GTH) pseudopotentials^76^, Grimme third-generation dispersion (D3) correction with zero damping^77^ and energy cutoffs of 600 and 60 Ry, respectively, for the plane-wave and Gaussian contributions to the basis set, as recommended in previous work^78^. Ensuring that each DFT calculation converges to the global-minimum energy is challenging for complex condensed-phase systems with a large number of molecules and partially broken bonds. Indeed, it is likely that the few large outliers observed in Supplementary Fig. 5 are DFT calculations that converged to a meta-stable electronic state. Fortunately, the fraction of these outliers is relatively low. Thus, these presumed meta-stable calculations do not meaningfully impede the MLIP from learning the dominant branch of convergence for the DFT calculations.

The overall spin multiplicity for each DFT calculation is constrained to a singlet state. Note that this is the spin for the entire box, not just a single molecule or radical. The assumption that a condensed-phase system does not accumulate an impactful amount of spin is effectively an infinite-system size approximation. This choice of spin multiplicity is consistent with previous studies that perform CP2K simulations of bulk systems containing radical species^79^. However, a singlet spin multiplicity for low-density gas phase or in vacuo calculations would not always be appropriate (for example, for radicals or molecules with partially formed bonds). The use of a singlet spin multiplicity may explain, in part, why ANI-1xnr performs poorly for *in vacuo* bond-breaking calculations (Extended Data Fig. 4).

Below is a detailed step-by-step description of the AL workflow (for a high-level overview, see Fig. 1):

1. Generate a bootstrap dataset (labelled with energies and forces) of 100 randomly generated periodic cells containing randomly placed and oriented small molecules including C_2_, H_2_, N_2_, O_2_, NH_3_, CH_4_, CO_2_, H_2_O and C_2_H_2_ with random composition
2. Train ensemble of ANI potentials to the current training dataset using eightfold (16 blocks) cross validation (14/1/1: train/validation/test split) scheme
3. Prepare for NR–AL sampling: build a new random box of small molecules with random size, density, placements orientations. Define a random schedule function for oscillating temperature (*T*) and density (*ρ*). Oscillating functional form is the same for temperature and density (see equations below), where *t* is time, ${t}_{\max }$ is a hyperparameter for the max time the simulation will run, and *T*_start_, *T*_end_, *T*_amp_, *ρ*_start_, *ρ*_end_, *ρ*_amp_ and *t*_per_ are randomly selected values within a pre-determined range (Supplementary Table 6):
  $$\begin{array}{rcl}T(t)&=&{T}_{{{{\rm{start}}}}}+\frac{t}{{t}_{\max }}({T}_{{{{\rm{end}}}}}-{T}_{{{{\rm{start}}}}})+{T}_{{{{\rm{amp}}}}}{\sin }^{2}\left(\frac{t}{{t}_{{{{\rm{per}}}}}}\right)\\ \rho (t)&=&{\rho }_{{{{\rm{start}}}}}+\frac{t}{{t}_{\max }}({\rho }_{{{{\rm{end}}}}}-{\rho }_{{{{\rm{start}}}}})+{\rho }_{{{{\rm{amp}}}}}{\sin }^{2}\left(\frac{t}{{t}_{{{{\rm{per}}}}}}\right)\end{array}$$
4. Run the NR MD simulation using forces from current AL-generation MLIP
5. Monitor energy and force uncertainty metrics every 5–50 MD steps (with an MD time step of 0.5 fs). If the uncertainty values exceed a pre-selected threshold value, end the simulation and add configuration to a new batch of structures. Otherwise, continue running MD for a maximum of 1 ns
6. Run DFT single-point calculations on the new batch of structures to obtain energy and force labels
7. Add new labelled data to the training dataset
8. Go back to step 2 and repeat until the MLIP converges. We define convergence as when MLIP-driven MD sampling simulations run for on the order of 50 ps on average. In other words, convergence is achieved when the MLIP is confident in all new MD simulations

### Resulting training dataset

After more than 50 iterations of AL, the resulting training dataset includes 26,650 simulation cells of atomic positions with corresponding DFT system energy and atomic forces. Two-dimensional visualizations of the local atomic environments present in the dataset (Fig. 2a–d) are generated using *t*-distributed stochastic neighbour embeddings^80^. Distributions of the system sizes, compositions and densities can be found in Supplementary Figs. 10–12, respectively. The average system size is 139 atoms. The vast majority (≈95%) of configurations in the training dataset have a density between 0.5 and 2.0 g cc^−1^. While the minimum density in the dataset is ≈0.03 g cc^−1^, less than 1% of the configurations in the dataset have a density less than 0.1 g cc^−1^, suggesting that ANI-1xnr should not be trusted for low-density gas-phase simulations or in vacuo calculations.

By cross-referencing the ANI-1xnr training dataset with the existing PubChem database^81^ for only CHNO molecules with ten or fewer CNO atoms, we conclude that the ANI-1xnr dataset contains 1,212 unique known PubChem molecules, or approximately 0.2% of the ≈555k PubChem CHNO molecules with ten or fewer CNO atoms. Supplementary Fig. 13 shows a histogram of the sizes of all molecules that are found in the ANI-1xnr dataset, which includes one molecule up to 145 atoms. The majority are small molecules of similar size or slightly larger than those from which the systems were initialized. There are many occurrences of molecules in the range of 10–90 atoms. The largest structures, ascertained by visual inspection, are graphene sheets. Furthermore, the 1,212 unique PubChem molecules only represent the simulation frames that were selected by the uncertainty estimate. Therefore, 1,212 should be considered a lower bound of molecules discovered during AL. There are probably many more molecules formed over all NR–AL sampling, which is estimated to be hundreds of nanoseconds of MD simulation time in aggregate.

To automate the extraction of common molecular entities that formed during the AL process, we developed a NetworkX-based package called MolFind. This Python software tool employs user prescribed cutoff distances for defining when two atoms are bonded or not and discovers clusters of atoms connected via bonds. The three-dimensional molecular architecture is partially captured through a graphical representation (that is, nodes and edges) of the bonding topology where atoms are nodes and bonds are edges. Graphs are encoded according to the open-source Python package called NetworkX^82^. The graphical representation and the NetworkX package enables (1) the counting of the number of topologically distinct molecular species in a simulation via a graph isomorphism check and (2) a comparison to known molecular entities with a specified topology. Previously, we tabulated a large database of known molecules and associated topologies by scraping the entirety of the PubChem database up to ten non-hydrogen atoms. The existence of a species in the database is not required for MolFind to extract a bonded atomic cluster but if found, it can affix a chemical/species name with the entity.

### Simulation details

All MD simulations in this study are performed with the NeuroChem package^67^ and the Atomic Simulation Environment^83^. The average computational speed of our Atomic Simulation Environment–NeuroChem MD simulations was approximately 50k atomic gradients per second on a single NVIDIA Titan V graphics processing unit (GPU). We acknowledge that a more optimized code, such as Large-scale Atomic/Molecular Massively Parallel Simulator (LAMMPS)^84^, would be noticeably more computationally efficient. For example, recent studies have demonstrated that highly optimized MLIPs within LAMMPS can obtain 1–10 million atomic gradients per second on a single NVIDIA A100 GPU^15,85^. However, because of the relatively small system sizes of our AL simulations (less than 500 atoms) and our case study simulations (no greater than 5,000 atoms), it was not necessary to fully optimize our computational performance by utilizing a code such as LAMMPS. To demonstrate that there is an opportunity to greatly improve our performance, we achieved 90k atomic gradients per second simply by increasing our simulation size to 25k atoms and, thereby, more efficiently utilizing the GPU.

### Carbon solid-phase nucleation

To investigate the formation process of diamond and graphene, MD simulations were performed for amorphous carbon under different densities. Three initial system structures with three different densities (0.5 g cc^−1^, 2.25 g cc^−1^ and 3.52 g cc^−1^) were generated by varying the simulation box length for a constant total number of carbon atoms of 5,000. The initial system structure was built with in-house code. First, the initial position for the first carbon atom in the simulation box was randomly selected. Then, random positions were proposed for each additional carbon atom. A proposed position was accepted only if the distance to all previous positions was larger than twice the van der Waals radius for carbon atoms (1.7 Å). This process was repeated until all 5,000 carbon atoms were inserted. Langevin dynamics were performed at a temperature of 2,500 K for 5 ns with step length of 0.5 fs. Coordinates and properties were recorded every 50 fs (100 time steps). Eight independent trajectories were run for each density to verify that the correct phase was identified from different starting structures. Different phases (diamond cubic, hexagonal diamond or graphene) in each snapshot were distinguished with the Open Visualization Tool^86^.

### Effect of oxygen on graphene ring formation

To investigate ring formation from acetylene, MD simulations were performed for eight different systems with varying O_2_/C_2_H_2_ ratios: (0.00, 0.08, 0.17, 0.22, 0.38, 0.50, 0.86 and 1.33). All systems contained 1,000 atoms, resulting in a range of 150–250 C_2_H_2_ molecules and 0–200 O_2_ molecules, depending on the O_2_/C_2_H_2_ ratio. To have a nearly identical density of 0.2 g cc^−1^ for each system, the box lengths ranged between 37 Å and 44 Å. The initial structures were generated with PackMol^87^. Next, the minimum-energy structure was obtained with the Limited-memory Broyden–Fletcher–Goldfarb–Shanno optimizer^88^. Then, Langevin dynamics simulations were run at 2,000 K for 10 ns with a 0.5 fs time step and a friction constant of 0.01. Snapshots and properties were recorded every 0.5 ps (1,000 time steps).

Ring structures of varying sizes were identified and counted with our in-house code MolFind. Considering that the distance between bonded atoms can fluctuate, a 0.02 Å buffer was utilized when scanning C–C bonds so that any pair of carbon atoms that has distance smaller than 1.72 Å (two times the covalent radius of carbon atom plus the buffer) were considered bonded. Similar buffers were also added when analysing other simulations.

### Comparison of biofuel additives

To investigate the effect of different fuel additives on ignition performance, MD simulations were performed for clean biofuel and biofuel with three different additives: ethanol, 2-butanol and MTBE. The biofuel composition, the number of additive molecules and the number of O_2_ molecules were the same as presented in Table 2 of the ReaxFF reference paper^50^. Each system consists of approximately 2,000 atoms. Initial structures were generated using Packmol such that the initial separation of all molecules was at least 2 Å. The initial density was 0.2 g cc^−1^ in all four cases, consistent with Chen et al. Langevin dynamics were run at a temperature of 100 K for 1 ps for relaxation. Then, the system temperature was gradually increased to 3,000 K at a 50 K ps^−1^ heating rate. After reaching the desired temperature of 3,000 K, the simulation was ran for an additional 10 ns. A fixed time step of 0.1 fs was utilized. The temperature, time step and heating profile were the same as those utilized by Chen et al.^50^ During the whole process (including relaxation and temperature ramping) snapshots and properties were recorded every 1 ps (10,000 time steps). Five independent trajectories were performed for each system to reduce uncertainty in species profiles.

ANI-1xnr was trained to BLYP reference calculations, whereas ReaxFF was primarily developed based on B3LYP calculations (supplemented with high-accuracy bond dissociation energy data). Since reaction rates are extremely sensitive to energy barriers, this difference in the DFT functional can lead to a substantial difference in overall reaction rates.

### Methane combustion

The methane combustion system was initialized with 100 CH_4_ molecules and 200 O_2_ molecules. All molecules were inserted using Packmol and ensuring that all molecules were separated by at least 2.0 Å. The cubic simulation box length was 37.60 Å, resulting in a density of 0.25 g cc^−1^. The temperature was initialized to 3,000 K by Maxwell–Boltzmann distribution. Langevin dynamics were run for 1 ns with a time step of 0.1 fs and with a friction constant of 0.01. The initial density, number of molecules, temperature and time step were consistent with Zeng et al.^24^. Snapshots and properties were recorded every 0.1 ps (1,000 time steps).

ANI-1xnr was trained to reference calculations computed with BLYP functional and TZV2P basis set, whereas Zeng et al. utilized the MN15 functional and 6-31G** basis set^24^. Since reaction rates are extremely sensitive to energy barriers, this difference in the DFT functional and basis set can lead to a substantial difference in overall reaction rates.

### Miller experiment

To investigate the ability to simulate complex organic system that involve biologically relevant molecules, an MD simulation was performed with a similar species composition to the Miller experiment. Packmol was utilized to randomly place 16 H_2_, 14 H_2_O, 14 CO, 14 NH_3_ and 14 CH_4_ in a cubic simulation box with edge lengths of 12.1 Å, resulting in a density of 1.067 g cc^−1^. The simulation was run with Langevin dynamics for over 4 ns with a time step of 0.25 fs. The temperature was linearly increased from 0 K to 300 K in the first 100 ps. Then, the temperature was linearly increased from 300 K to 2,500 K in the next 100 ps. The temperature was then maintained at 2,500 K for 4,000 ps. The system was then cooled from 2,500 K to 300 K over the final 200 ps. Snapshots and properties were recorded every 12.5 fs (50 time steps).

Although some differences exist between our mechanism and those reported in previous simulation studies, this is not surprising considering not only the difference in levels of theory (that is, Hartree–Fock versus DFT versus MLIP), but also the difference in the simulation methodologies (that is, our Miller simulation did not utilize a ‘piston’ nor induce an electric field). For this reason, we further validate our Miller experiment results by comparing the ANI-1xnr energies and forces directly with DFT calculations along the MD trajectory. These validation results are provided in Supplementary Fig. 4.

## Online content

Any methods, additional references, Nature Portfolio reporting summaries, source data, extended data, supplementary information, acknowledgements, peer review information; details of author contributions and competing interests; and statements of data and code availability are available at 10.1038/s41557-023-01427-3.

### Supplementary information

Supplementary Information Supplementary Tables 1–6 and Figs. 1–13.

Supplementary Data 1 Initial and final Cartesian coordinates for simulations performed in each case study.

### Source data

Source Data Fig. 4 Effect of oxygen on graphene ring formation simulation results for ANI-1xnr.

Source Data Fig. 5 Biofuel additive simulation results for ANI-1xnr.

Source Data Fig. 6 Methane combustion simulation results for ANI-1xnr.

## Figures and Tables

**Extended Data Fig. 2:**
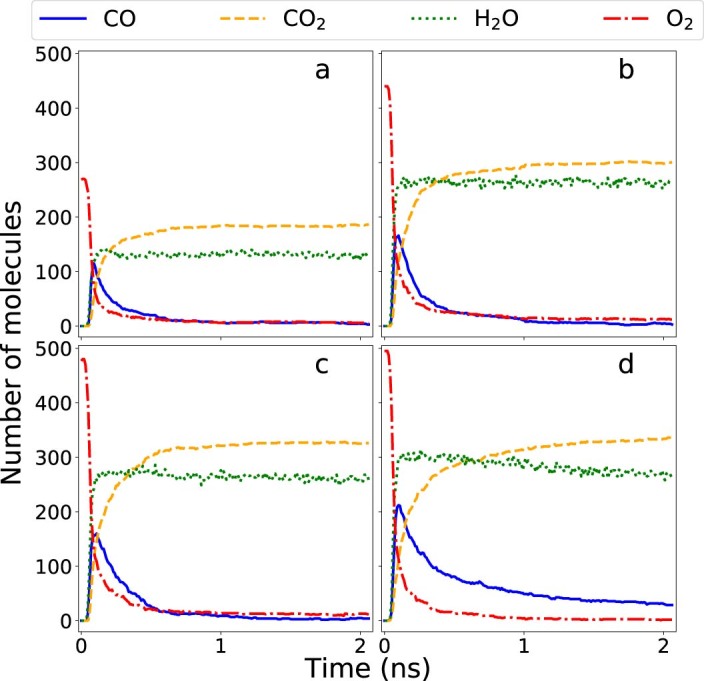
Biofuel additive simulation results for ANI-1xnr over the entire simulation. Tracking plot of O_2_ and major products (CO, CO_2_, and H_2_O) for biofuel simulations: (a) biofuel+O_2_ (b) biofuel+O_2_ with ethanol additive (c) biofuel+O_2_ with 2-butanol additive (d) biofuel+O_2_ with MTBE additive.

**Extended Data Fig. 6:**
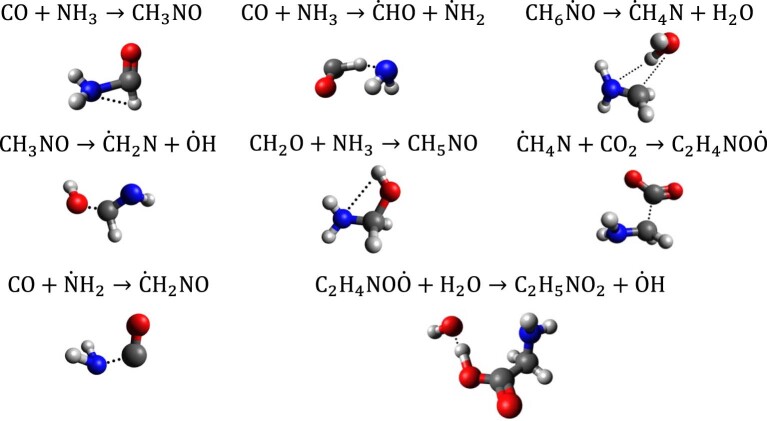
Three-dimensional structures of reactions discovered by ANI-1xnr in Miller experiment simulation. Structures are snapshots extracted directly from the MD simulation trajectory, providing visual confirmation that the reaction pathways are physically meaningful.

**Extended Data Fig. 7:**
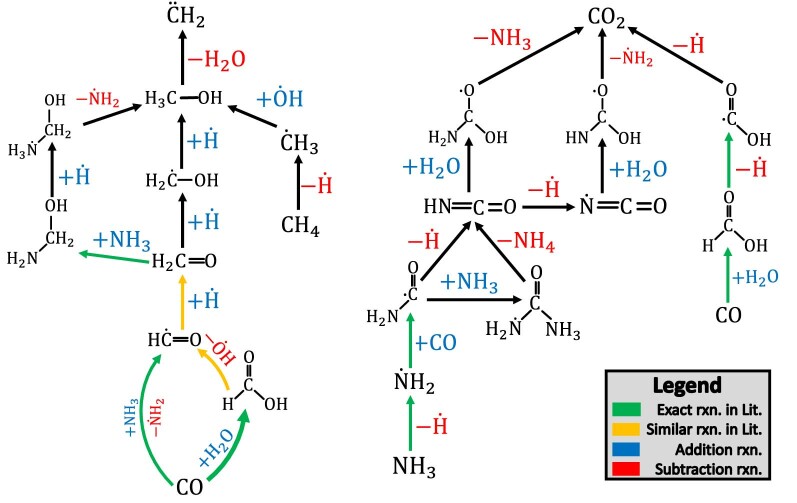
Reaction mechanisms for formation of key intermediates (CH_2_ and CO_2_) from initial reactants (NH_3_, CO, CH_4_, H_2_O) in Miller simulation. Green arrows denote reactions previously identified by Wang et al. or Saitta and Saija. Orange arrows denote reactions that have closely-related reactions in Wang et al. or Saitta and Saija.

**Extended Data Fig. 8:**
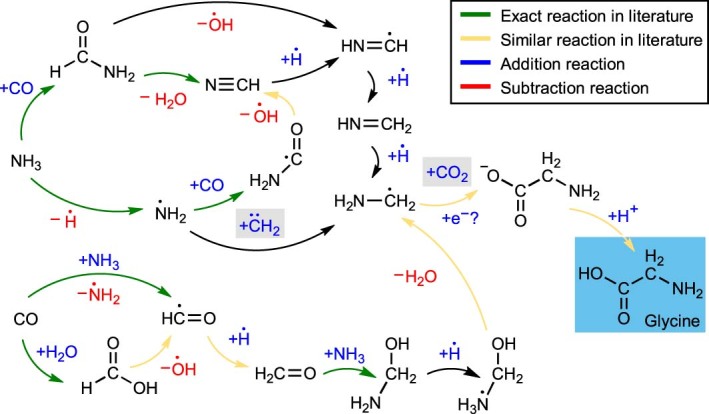
Alternative mechanism for the formation of glycine in the Miller experiment simulation. In this pathway, the final step to form glycine involves H-abstraction from H_3_O, which chemical intuition would label as a cationic species (H_3_O^+^). The penultimate species (${{{{\rm{C}}}}}_{2}{{{{\rm{H}}}}}_{4}{{{{\rm{NO}}}}}_{2}^{-}$) formed prior to glycine, therefore, cannot be unambiguously labeled as an anion or a radical. The uncertainty regarding the ionic nature of this mechanism illustrates an issue with electron-agnostic MLIPs, like ANI-1xnr. The depiction of bond orders, charges on ions, and radical species is based simply on chemical intuition, since ANI-1xnr does not provide explicit bonding, orbital, or electronic information.

## Data Availability

The ANI-1xnr training dataset is publicly available at 10.6084/m9.figshare.22814579. Initial and final structures for case study simulations are provided as Supplementary Data files. Details are provided in the corresponding section in Methods. Source data are provided with this paper.

## Extended data

**Extended Data Table 1:** Comparison of optimized crystal lattice constants (*a*, *b*, *c*) for diamond and graphite phases

Crystal	Model	*a* (Å)	*b* (Å)	*c* (Å)
Diamond	ANI-1xnr	3.58	3.58	3.58
	ANI-1xnr(lr)	3.66	3.66	3.66
	ANI-2x	3.75	3.75	3.64
	Exp.	3.57	3.57	3.57
Graphite	ANI-1xnr	2.47	2.47	6.24
	ANI-1xnr(lr)	2.46	2.46	6.56
	ANI-2x	2.44	2.44	10.0
	Exp.	2.46	2.46	6.71

Comparison between ANI-1xnr, ANI-1xnr(lr), ANI-2x and experiment (Exp.). ANI-1xnr reproduces the diamond cubic lattice constants, the *a* and *b* lattice constants in graphite, and the non-orthogonal experimental cell angles for both diamond and graphite (see Supplementary Table 1). However, the *c* lattice constant in graphite (along the direction of *π*-*π* stacking) is predicted with a relatively large error of 0.47 Å. This relatively large error is likely due to ANI-1xnr being a short-range potential, while long-range dispersion interactions are important for *π*-*π* stacking. We also trained an MLIP with a longer-range local cutoff (5.5/4.5 Å) than the original ANI-1xnr potential (5.2/3.5 Å), called ANI-1xnr(lr). ANI-1xnr(lr) performs significantly better than the original ANI-1xnr on the *c* lattice parameter. However, ANI-1xnr(lr) performs worse on diamond cubic. A possible explanation for this reduction in accuracy is that larger cutoffs reduce the resolution of the local atomic descriptors, which can affect accuracy in dense chemical environments. This shortcoming could be resolved by increasing the number of symmetry functions on the longer-range MLIP, but this would greatly impact the computational speed of the potential. A more optimal solution would be to add an explicit dispersion correction to ANI-1xnr that captures long-range interactions while maintaining an accurate description of the local environment. We also compare the ANI-1xnr lattice constants with those from ANI-2x^18^, a model explicitly trained to small organic molecules as a baseline. ANI-2x performs poorly at predicting the lattice constants for both diamond cubic and graphite. This result is expected since the dataset used to train ANI-2x does not contain any structures similar to either of these systems. Furthermore, in contrast to the ANI-2x dataset reference calculations, the reference calculations used for building the ANI-1xnr dataset include dispersion corrections (see the Methods section for details), which are essential to reproduce the *c* lattice parameter in graphite.

**Extended Data Table 2:** Comparison between ANI-1xnr and ReaxFF ignition delay time (IDT) and O_2_ consumption for clean biofuel and biofuel with each of the three additives

System	Ignition delay time (ps)		O_2_ consumption (%)	
	ANI-1xnr	ReaxFF	ANI-1xnr (t = 0.07 ns)	ReaxFF (t = 2 ns)
Clean biofuel	55	239	49.0%	48.5%
Ethanol additive	45	126	58.6%	49.21%
2-butanol additive	46	110	57.5%	73.33%
MTBE additive	45	92	57.4%	70.3%

Ignition delay time is defined as the average time that at least five molecules of CO, CO_2_, and H_2_O are produced. While the reduction in IDT is not as pronounced for ANI-1xnr compared to ReaxFF, IDTs are highly sensitive as to how the system is initialized and to how ignition is defined (see Supplementary Figure 1). O_2_ consumption is compared at 0.07 ns for ANI-1xnr and at 2 ns for ReaxFF, that is, the time that the O_2_ consumption for the clean biofuel is approximately equal for both models. After the first 0.07 ns of ANI-1xnr simulation, 50% of O_2_ was consumed in the pure biofuel system, while systems with additives consumed around 60% of O_2_ (for O_2_ consumption plots, see Supplementary Figure 2). By contrast, in the ReaxFF simulations both the clean biofuel and ethanol additive systems consumed around 50% of O_2_ after 2 ns, while the 2-butanol additive and MTBE additive systems consumed about 70% of O_2_. The overall rate of fuel and O_2_ consumption is considerably faster for ANI-1xnr compared with ReaxFF. Specifically, for all four cases, nearly all of the O_2_ was consumed in the first 0.3 ns with ANI-1xnr, while there was still 20%-50% unconsumed O_2_ after 2 ns with ReaxFF (for tracking plots including the entire 2 ns simulation, see Extended Data Figure 2). The discrepancy in overall reaction rates between ANI-1xnr and ReaxFF is likely due to a difference in the underlying QM approach used to build each model (see Methods for details).

## Code availability

The ANI-1xnr model can be found at https://github.com/atomistic-ml/ani-1xnr/.
