# Supplementary material for: Exploring the frontiers of condensed-phase chemistry with a general reactive machine learning potential
Source: Nat Chem. 2024 Mar 7;16(5):727–34. doi: 10.1038/s41557-023-01427-3 (PMC11087274; doi:10.1038/s41557-023-01427-3)
Supplement: Supplementary file 1 — Supplementary Tables 1–6 and Figs. 1–13. [file 41557_2023_1427_MOESM1_ESM.pdf]

nature chemistry

Article

https://doi.org/10.1038/s41557-023-01427-3

Exploring the frontiers of condensed-phase chemistry with a general reactive machine learning potential

In the format provided by the authors and unedited

Contents

1 Comparison of optimized crystal angles for diamond and graphite phases 2
2 Ignition delay time (IDT) for biofuel simulations 3
3 Plot of major species of the methane combustion simulations 5
4 Validation of Miller Experiment simuluation 6
5 Settings and hyperparameters of the ANI-1xnr model 7
6 Model performance against held-out test dataset 8
7 Ensemble member performance against held-out test dataset 9
8 Energy correlation plot on the held-out test dataset 10
9 Force correlation plot on the held-out test dataset 11
10 Energy correlation plots for all ensemble members on the held-out test datasets 12
11 Force correlation plots for all ensemble members on the held-out test datasets 13
12 Validation of conservation of energy for ANI-1xnr 14
13 Parameters for nanoreactor oscillations in temperature and density 15
14 System size distribution of the ANI-1xnr training dataset 16
15 System composition of all systems in the training dataset 17
16 Mass density distribution of the ANI-1xnr training dataset 18
17 Molecule size distribution of the ANI-1xnr training dataset 19
References 20

1 Comparison of optimized crystal angles for diamond and graphite phases

Crystal	Model	α (^{o})	β (^{o})	γ (^{o})
Diamond	ANI-1xnr	90.0	90.0	90.0
	ANI-1xnr(lr)	90.0	90.0	90.0
	ANI-2x	90.0	90.0	90.0
	Exp.	90.0	90.0	90.0
Graphite	ANI-1xnr	90.0	90.0	120.
	ANI-1xnr(lr)	90.0	90.0	120.
	ANI-2x	90.4	89.7	120.
	Exp.	90.0	90.0	120.

Table 1. Optimized crystal angles (α, β, γ) for diamond and graphite phases. Comparison between ANI-1xnr, ANI-1xnr(lr), ANI-2x and experiment (Exp.).

2 Ignition delay time (IDT) for biofuel simulations

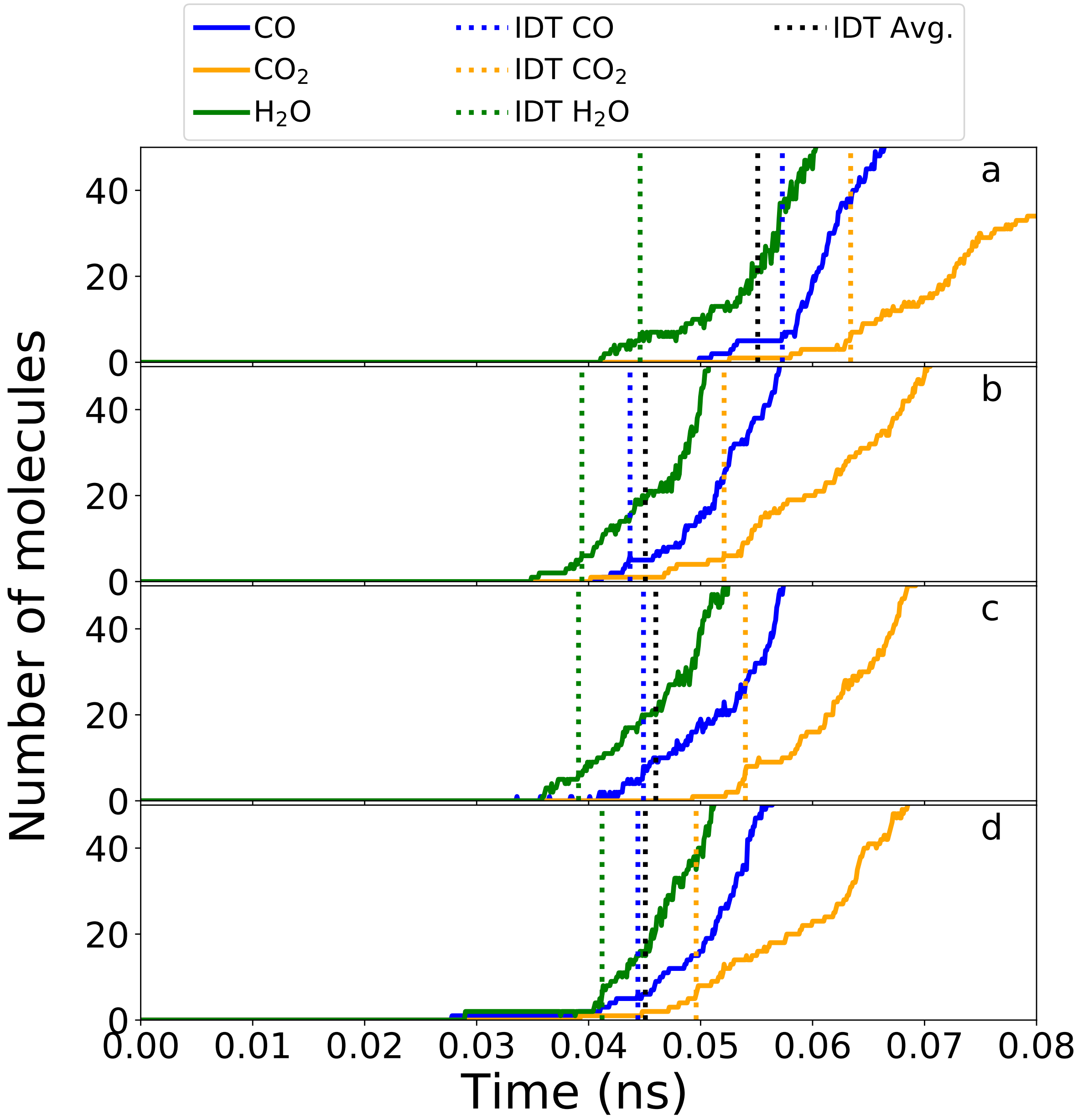

Figure 1. Ignition delay time (IDT) for biofuel simulations based on each of the major products CO, CO_2, and H_2O. To remove anomalies when only a single molecule is produced significantly prior to "true" ignition, we define IDT as the earliest time that at least five molecules of a given product are produced. The manuscript uses the average IDT value between CO, CO_2, and H_2O. (a) biofuel+O_2 system (b) biofuel with ethanol+O_2 (c) biofuel with 2-butanol+O_2 (d) biofuel with MTBE+O_2.

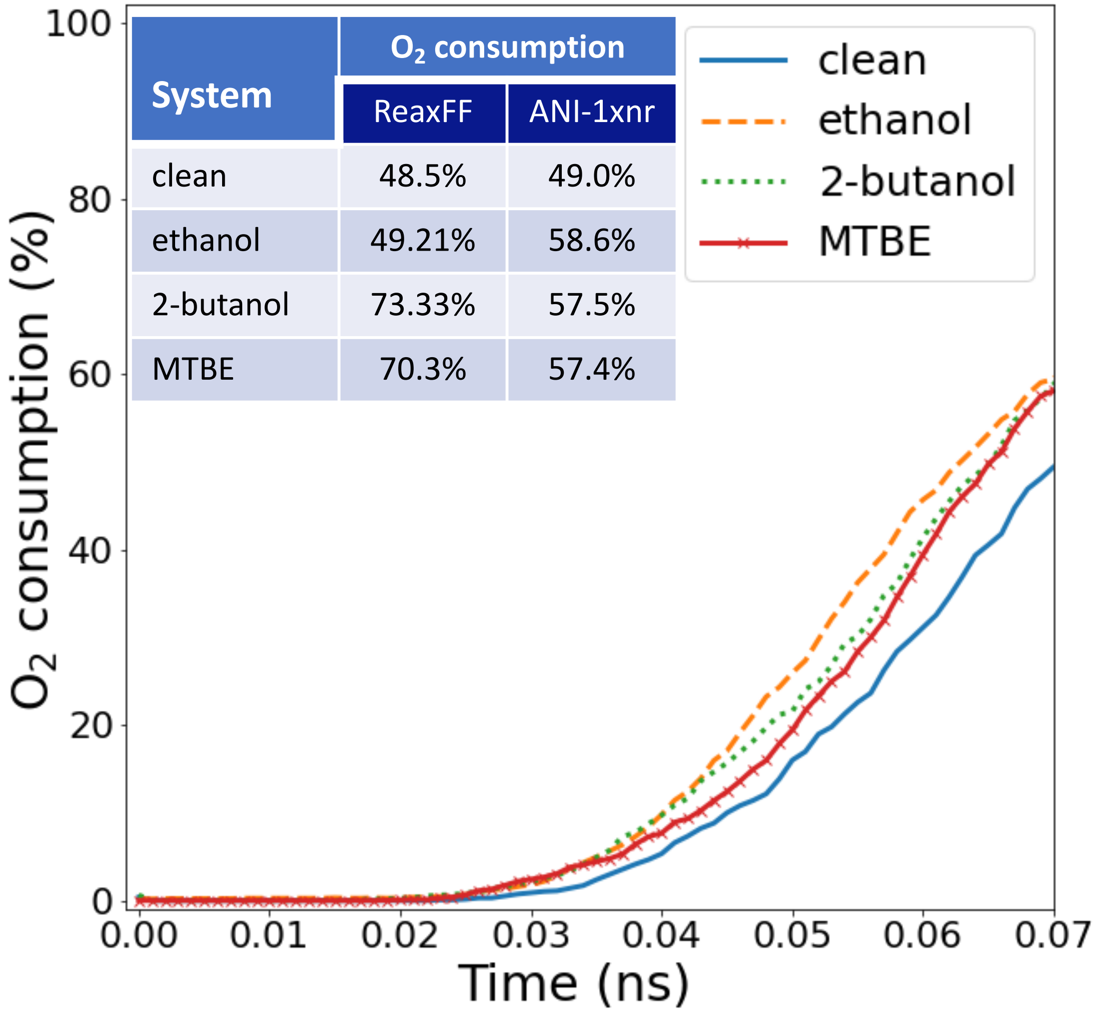

Figure 2. O_2 consumption (%) during combustion for clean biofuel compared with three different fuel additives, namely, ethanol, 2-butanol, and MTBE. Insert compares O_2 consumption for ANI-1xnr (at 0.07 ns) with ReaxFF (at 2 ns). Curves are smoothed by averaging over 5 independent trajectories.

3 Plot of major species of the methane combustion simulations

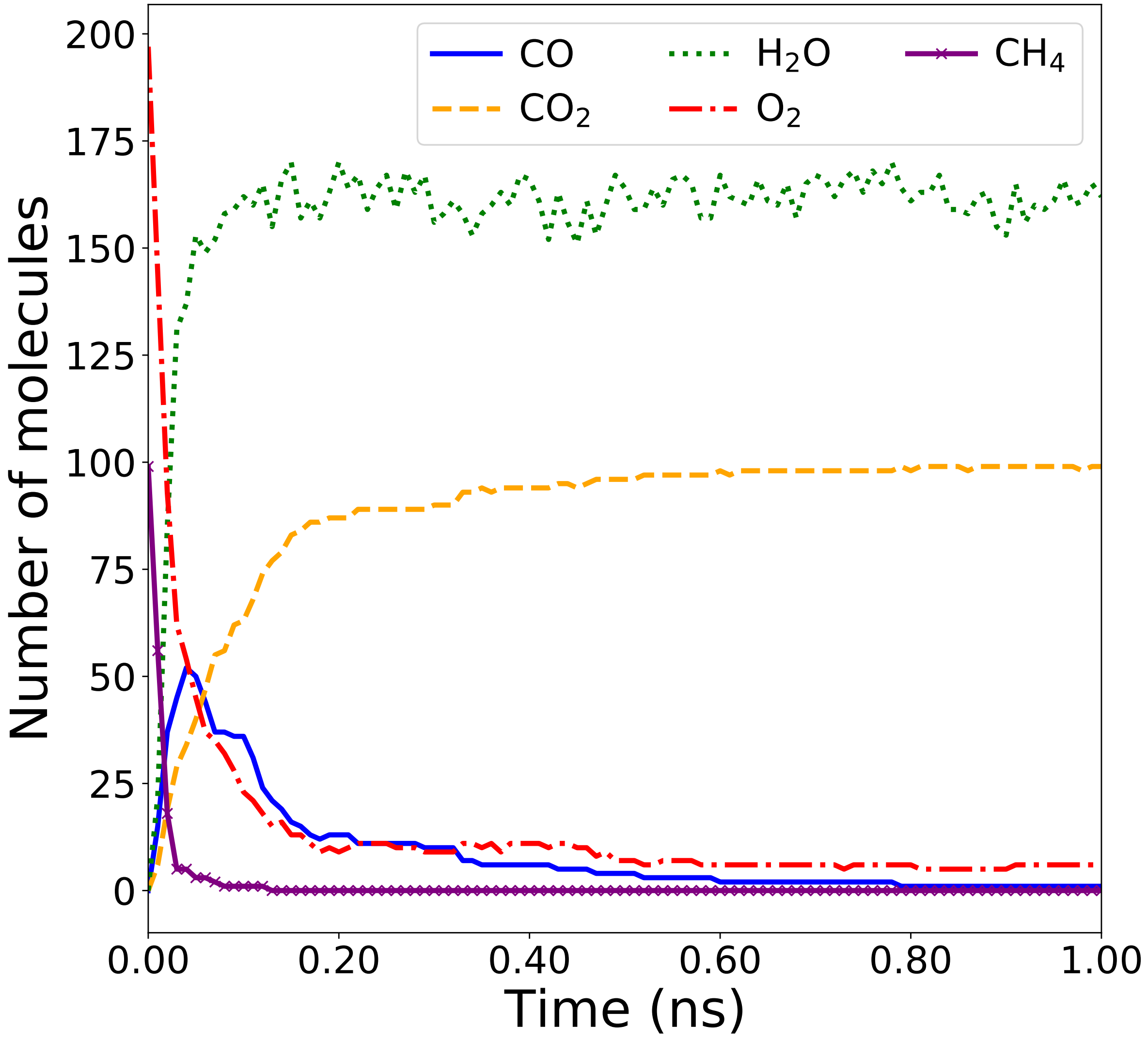

Figure 3. Tracking plot of O_2, CH_4, and major products (CO, CO_2, and H_2O) for the entire methane combustion simulation.

4 Validation of Miller Experiment simulation

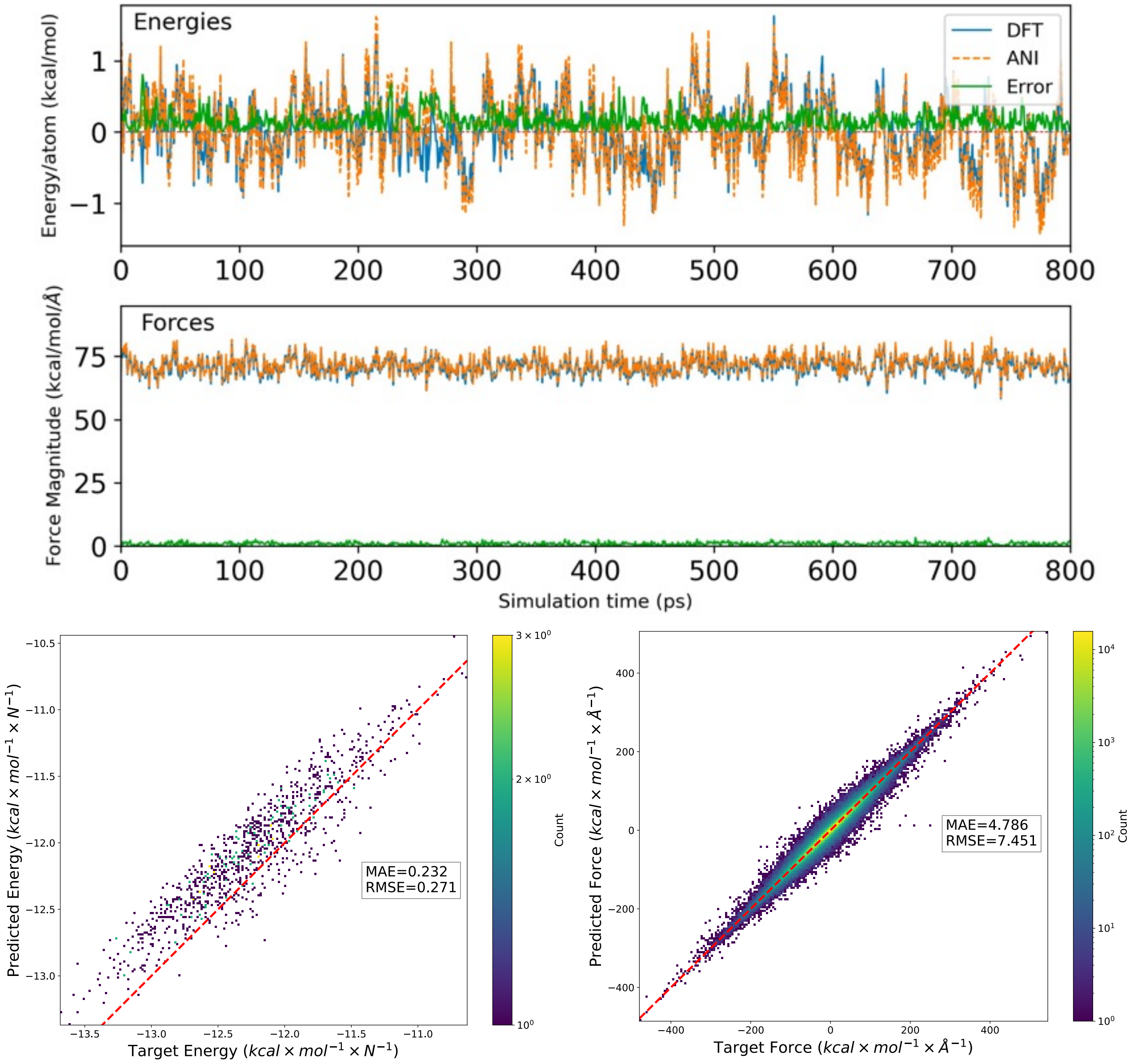

Figure 4. Validation of Miller Experiment simulation. Comparison between DFT energies and forces with ANI-1xnr for the first 800 ps. Data were not used in training of ANI-1xnr. ANI-1xnr is consistent with the underlying reference DFT along this simulation trajectory. Further validation of ANI-1xnr would require an extensive investigation into the accuracy of our DFT approach for the complete reaction network of this system.

5 Settings and hyperparameters of the ANI-1xnr model

The ANI neural networks used in this work were implemented in the NeuroChem C++/CUDA software package. A batch size of 32 was used while training the ANI-1xnr model. A weight of 1.0 was used on both the energy and force loss term. Learning rate annealing was used during training, starting at a learning rate of 0.001 and converging at a learning rate of 0.00001. The ADAM update algorithm is used during training[1]. The network architecture is provided in Table 2. The symmetry function parameters are provided in Table 3.

	H		C		N		O	
Layer ID	Nodes	Activation	Nodes	Activation	Nodes	Activation	Nodes	Activation
1	256	CELU	224	CELU	192	CELU	192	CELU
2	192	CELU	190	CELU	160	CELU	160	CELU
3	160	CELU	160	CELU	128	CELU	128	CELU
4	1	Linear	1	Linear	1	Linear	1	Linear

Table 2. ANI-1xnr neural network architecture

Radial Cutoff (Radial) (Å)	5.2
Radial Cutoff (Angular) (Å)	3.5
Radial Eta ($Å^{-2}$)	65.7
Radial Shift (Å)	0.500000,0.646875,0.793750,0.940625, 1.087500,1.234375,1.381250,1.528125, 1.675000,1.821875,1.968750,2.115625, 2.262500,2.409375,2.556250,2.703125, 2.850000,2.996875,3.143750,3.290625, 3.437500,3.584375,3.731250,3.878125, 4.025000,4.171875,4.318750,4.465625, 4.612500,4.759375,4.906250,5.053125
Angular Zeta (-)	14.1
Angular Angular Shift (rad.)	0.39269908,1.1780972, 1.9634954,2.7488936
Angular Eta ($Å^{-2}$)	10.1
Angular Radial Shift (rad.)	0.500,0.875,1.250,1.625, 2.000,2.375,2.750,3.125

Table 3. ANI-1xnr symmetery function parameters

6 Model performance against held-out test dataset

Property	RMSE	MAE
Energy (per-atom) $(\mathrm{kcal}\cdot\mathrm{mol}^{-1}\cdot N^{-1})$	0.43 ± 0.22	0.1756 ± 0.0061
Energy $(\mathrm{kcal}\cdot\mathrm{mol}^{-1})$	34.1 ± 6.5	22.04 ± 0.56
Force $(\mathrm{kcal}\cdot\mathrm{mol}^{-1}\cdot \mathrm{\AA}^{-1})$	10.34 ± 0.25	6.306 ± 0.074

Table 4. Model performance against held-out test dataset. Root-mean-squared-error (RMSE) and mean-absolute-error (MAE) are reported as the average of eight ensemble models with the corresponding standard deviation. Energy errors are reported both as unnormalized and per-atom normalized values.

7 Ensemble member performance against held-out test dataset

Property	RMSE	MAE
Ensemble member 1		
Energy (per-atom) ($kcal \cdot mol^{-1} \cdot N^{-1}$)	0.37	0.178
Energy ($kcal \cdot mol^{-1}$)	31.7	21.8
Force ($kcal \cdot mol^{-1} \cdot Å^{-1}$)	10.19	6.24
Ensemble member 2		
Energy (per-atom) ($kcal \cdot mol^{-1} \cdot N^{-1}$)	0.23	0.173
Energy ($kcal \cdot mol^{-1}$)	31.2	21.9
Force ($kcal \cdot mol^{-1} \cdot Å^{-1}$)	10.39	6.33
Ensemble member 3		
Energy (per-atom) ($kcal \cdot mol^{-1} \cdot N^{-1}$)	0.26	0.170
Energy ($kcal \cdot mol^{-1}$)	31.4	21.7
Force ($kcal \cdot mol^{-1} \cdot Å^{-1}$)	10.39	6.38
Ensemble member 4		
Energy (per-atom) ($kcal \cdot mol^{-1} \cdot N^{-1}$)	0.22	0.169
Energy ($kcal \cdot mol^{-1}$)	30.6	21.8
Force ($kcal \cdot mol^{-1} \cdot Å^{-1}$)	9.95	6.19
Ensemble member 5		
Energy (per-atom) ($kcal \cdot mol^{-1} \cdot N^{-1}$)	0.45	0.177
Energy ($kcal \cdot mol^{-1}$)	32.5	21.3
Force ($kcal \cdot mol^{-1} \cdot Å^{-1}$)	10.17	6.25
Ensemble member 6		
Energy (per-atom) ($kcal \cdot mol^{-1} \cdot N^{-1}$)	0.23	0.175
Energy ($kcal \cdot mol^{-1}$)	32.8	23.2
Force ($kcal \cdot mol^{-1} \cdot Å^{-1}$)	10.21	6.33
Ensemble member 7		
Energy (per-atom) ($kcal \cdot mol^{-1} \cdot N^{-1}$)	0.24	0.172
Energy ($kcal \cdot mol^{-1}$)	31.4	22.1
Force ($kcal \cdot mol^{-1} \cdot Å^{-1}$)	10.57	6.43
Ensemble member 8		
Energy (per-atom) ($kcal \cdot mol^{-1} \cdot N^{-1}$)	0.91	0.189
Energy ($kcal \cdot mol^{-1}$)	51.2	22.6
Force ($kcal \cdot mol^{-1} \cdot Å^{-1}$)	10.81	6.29

Table 5. Ensemble member performance against held-out test dataset. Root-mean-squared-error (RMSE) and mean-absolute-error (MAE) are reported for each individual ensemble member. Energy errors are reported both as unnormalized and per-atom normalized values. Recall that each ensemble member is evaluated against a different held-out dataset. Therefore, although ensemble member 8 has a higher energy RMSE (with an MAE that is similar to other ensemble members), this appears to be the result of some large outliers, potentially from some poor DFT results (see Figure 7).

8 Energy correlation plot on the held-out test dataset

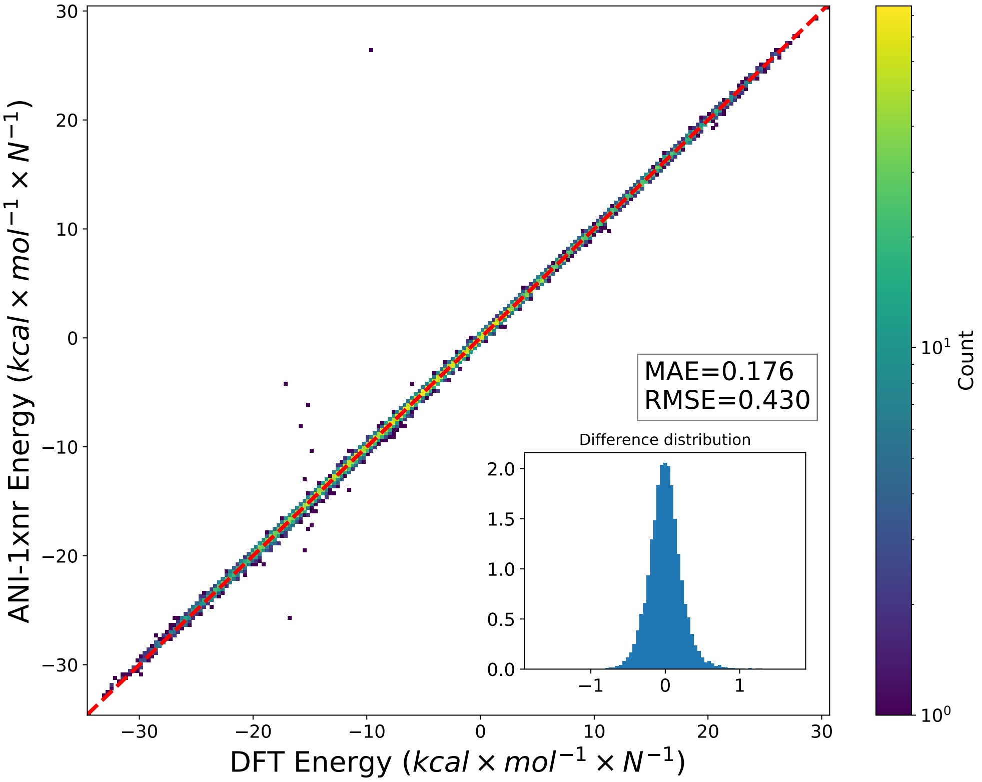

Figure 5. Energy correlation plot. Root-mean-squared-error (RMSE) and mean-absolute-error (MAE) are reported as the average of eight ensemble models against a held-out test dataset.

9 Force correlation plot on the held-out test dataset

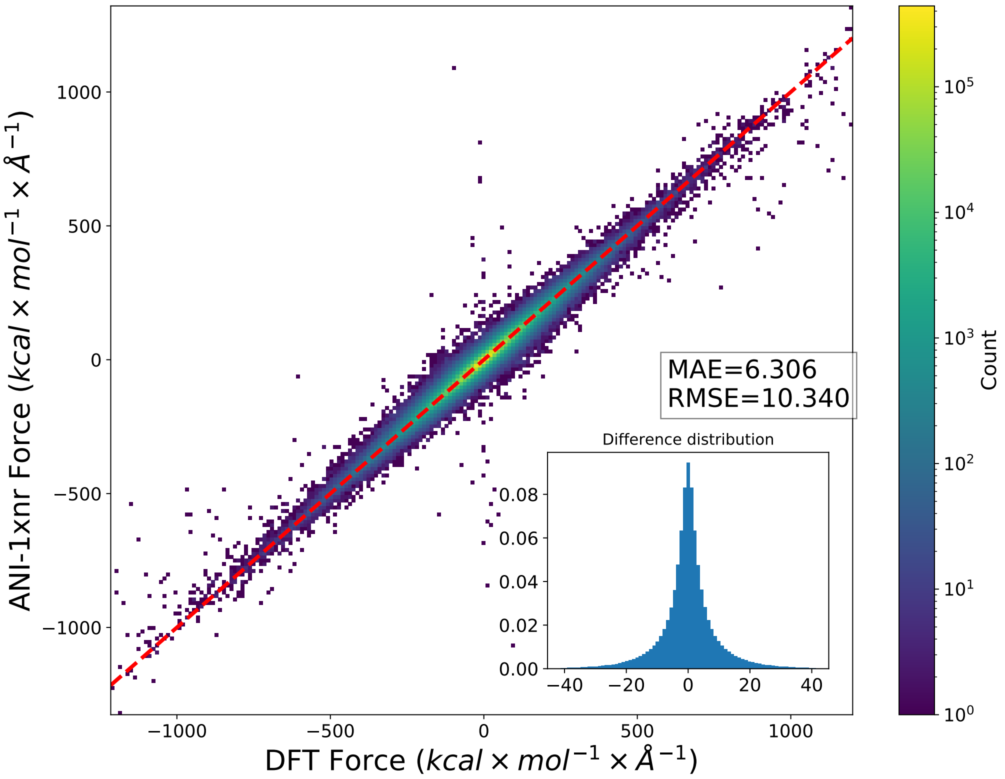

Figure 6. Force correlation plot. Root-mean-squared-error (RMSE) and mean-absolute-error (MAE) are reported as the average of eight ensemble models against a held-out test dataset.

10 Energy correlation plots for all ensemble members on the held-out test datasets

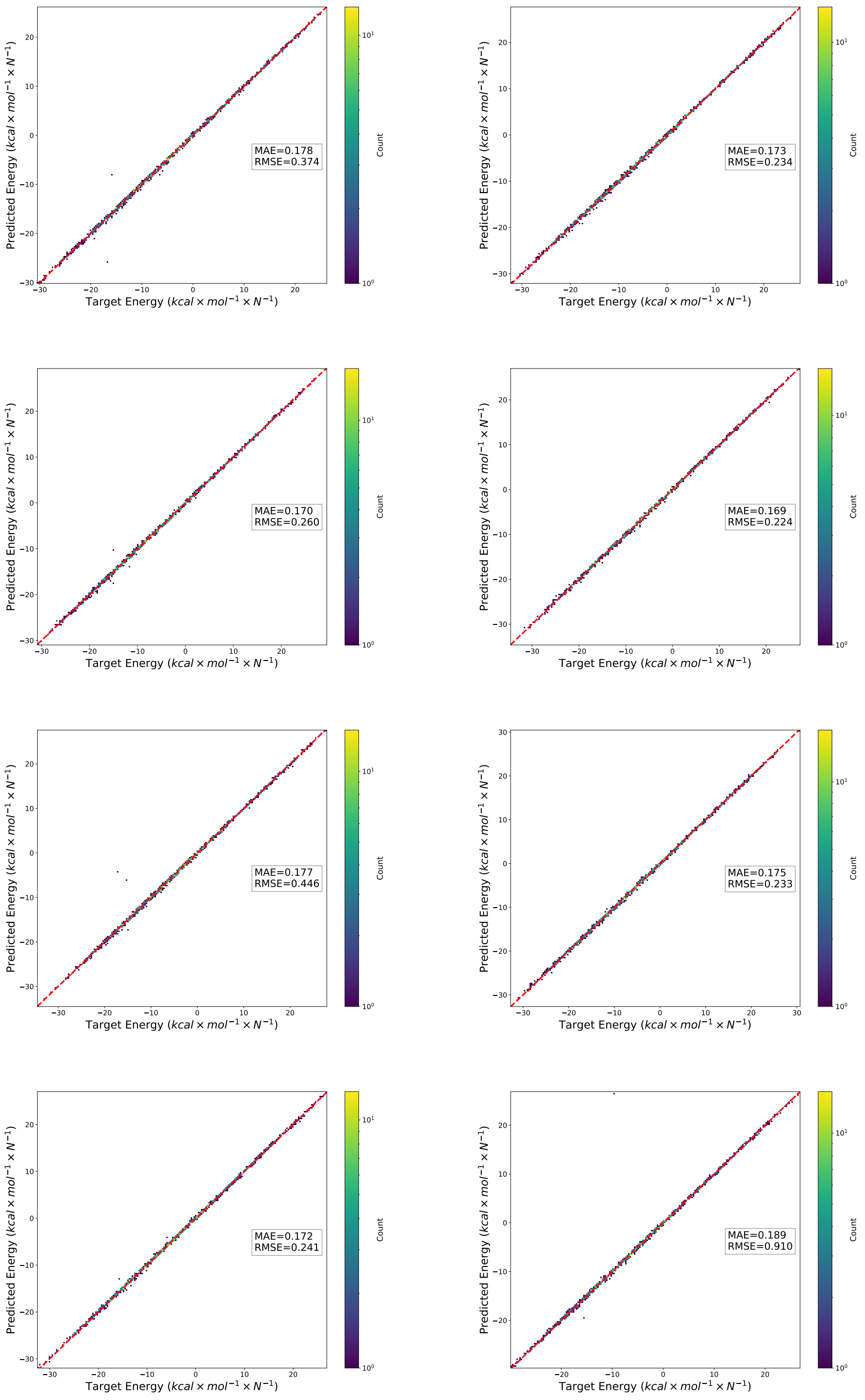

Figure 7. Energy correlation plots for all ensemble members (members 1-8 from top-left to bottom-right). Root-mean-squared-error (RMSE) and mean-absolute-error (MAE) are reported for each individual ensemble member. Recall that each ensemble member is evaluated against a different held-out test dataset. Therefore, although ensemble member 8 has a higher energy RMSE (with an MAE that is similar to other ensemble members), this appears to be the result of some large outliers, potentially from some poor DFT calculations.

11 Force correlation plots for all ensemble members on the held-out test datasets

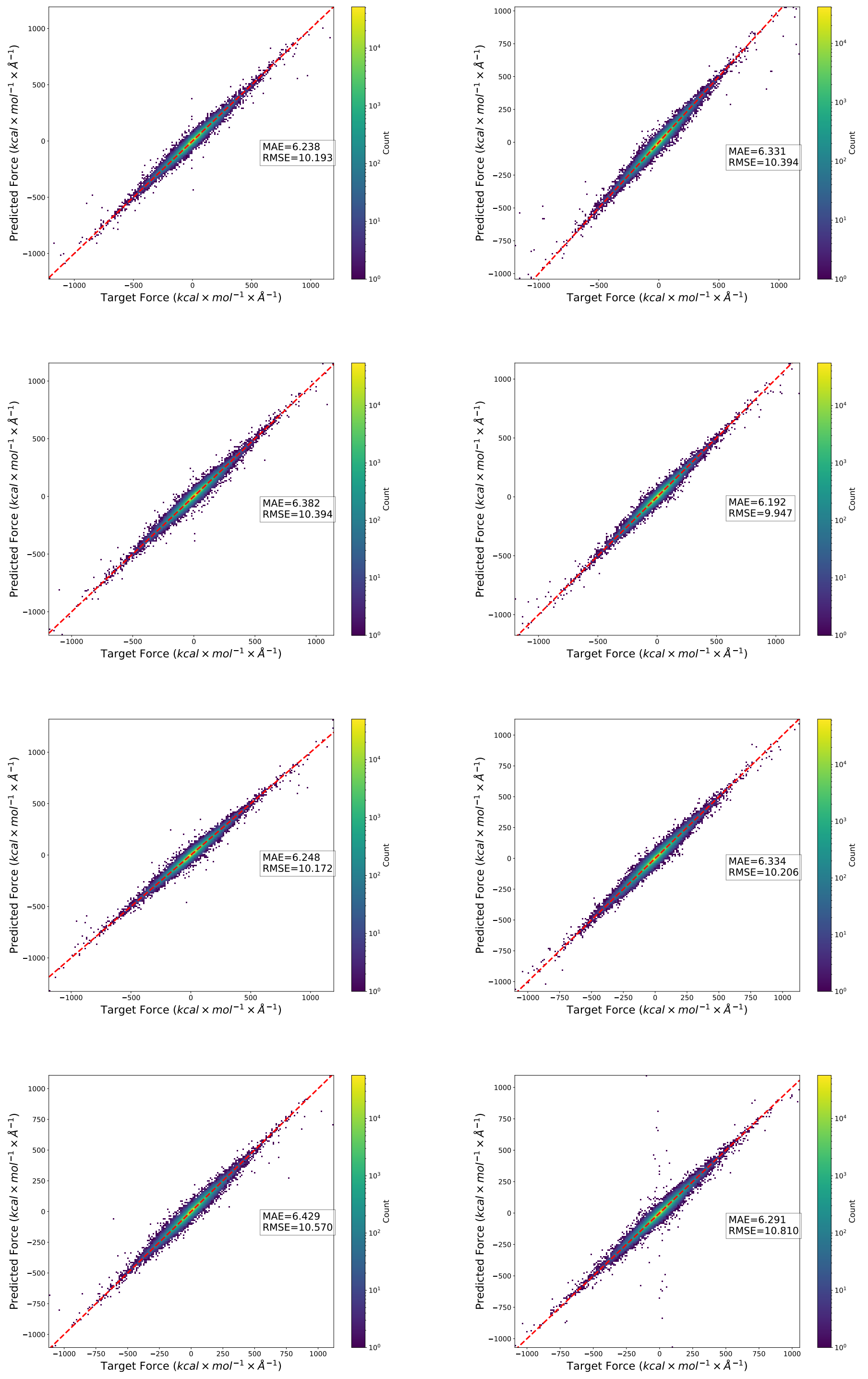

Figure 8. Force correlation plots for all ensemble members (members 1-8 from top-left to bottom-right). Root-mean-squared-error (RMSE) and mean-absolute-error (MAE) are reported for each individual ensemble member. Recall that each ensemble member is evaluated against a different held-out test dataset.

12 Validation of conservation of energy for ANI-1xnr

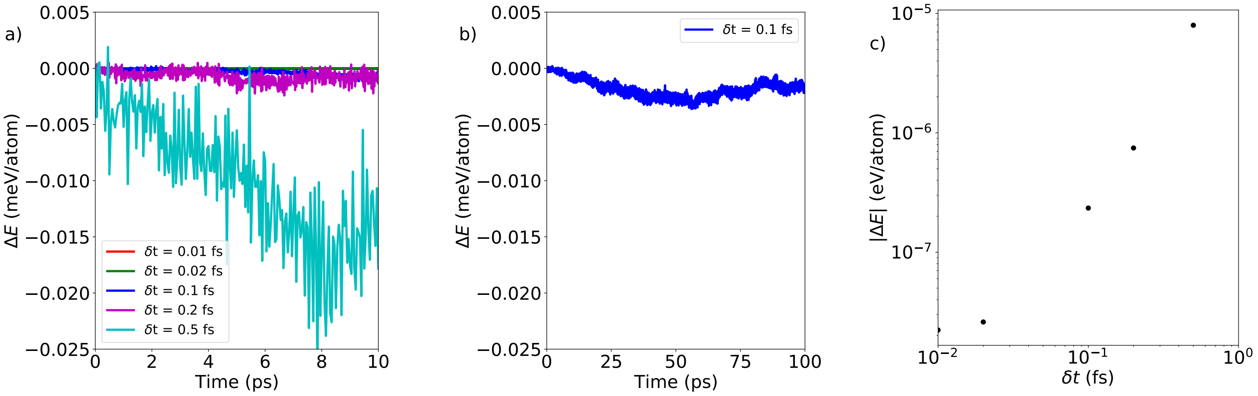

Figure 9. Validation of conservation of energy for ANI-1xnr. All results correspond to NVE simulations for the CH_4+O_2 system. Panel a) compares the energy drift over a 10 ps simulation with different time-steps. This figure justifies the choice of a 0.1 fs time-step for this system. Panel b) presents the energy drift for a 100 ps simulation with a 0.1 fs time-step. The average energy drift with this time-step is approximately -7.6 $\times$ 10 $^{-8}$ eV/ps/atom. Panel c) demonstrates that the average energy fluctuations scale approximately linearly on a log-log plot with respect to time-step size. Compare to Figure 5 in de Oca Zapiain et al.[2]

13 Parameters for nanoreactor oscillations in temperature and density

Parameter	Range
T_{start}	1000 - 3000 K
T_{end}	100 - 2000 K
T_{amp}	0 - 2000 K
ρ_{start}	0.1 - 2 g/cc
ρ_{end}	0.5 - 2 g/cc
ρ_{amp}	0 - 0.75 g/cc
t_{per}	T: 2 - 50 ps; ρ: 0.5 - 50 ps

Table 6. Parameters for nanoreactor oscillations in temperature and density.

14 System size distribution of the ANI-1xnr training dataset

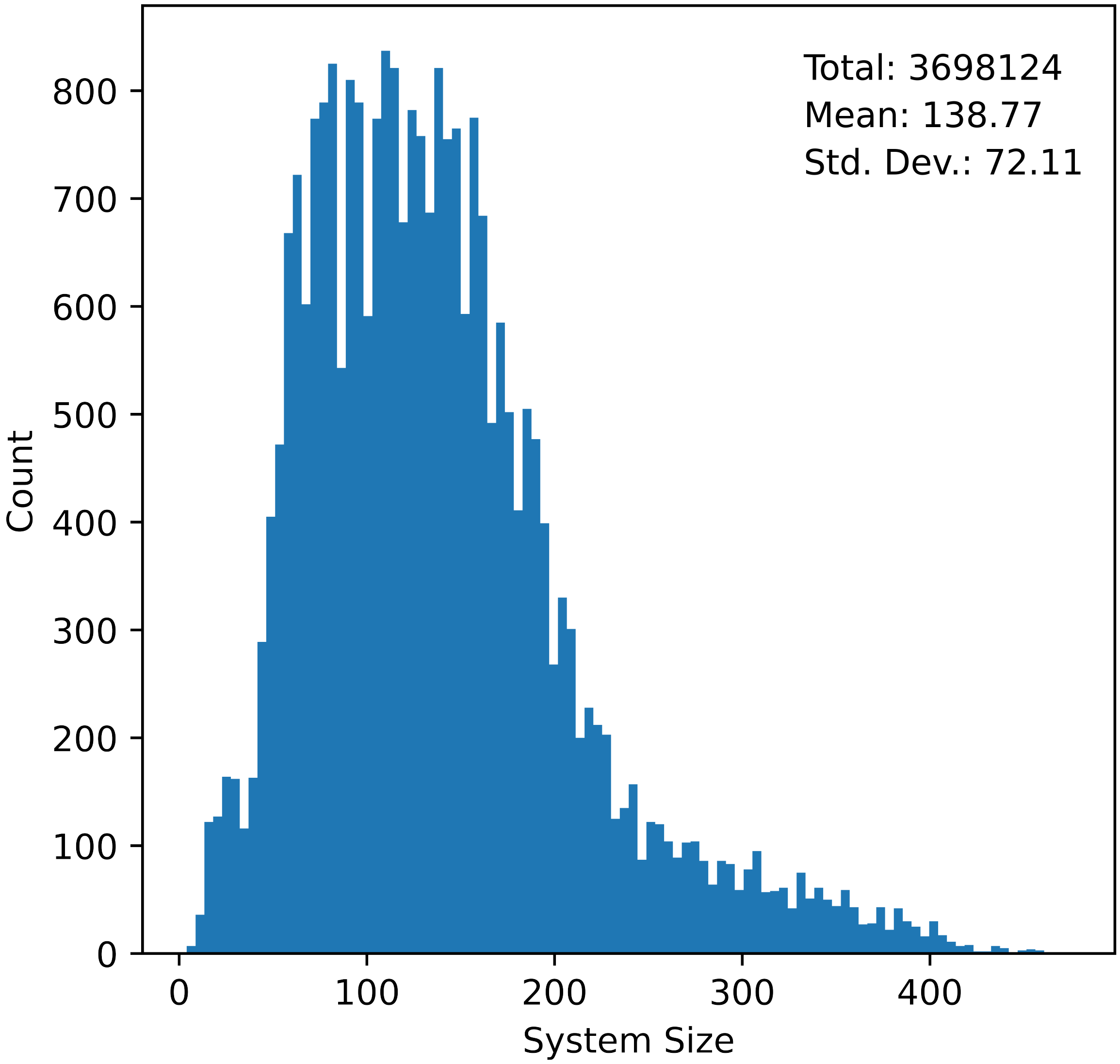

Figure 10. Histogram of the system size (i.e., number of atoms) per system in the ANI-1xnr training dataset.

15 System composition of all systems in the training dataset

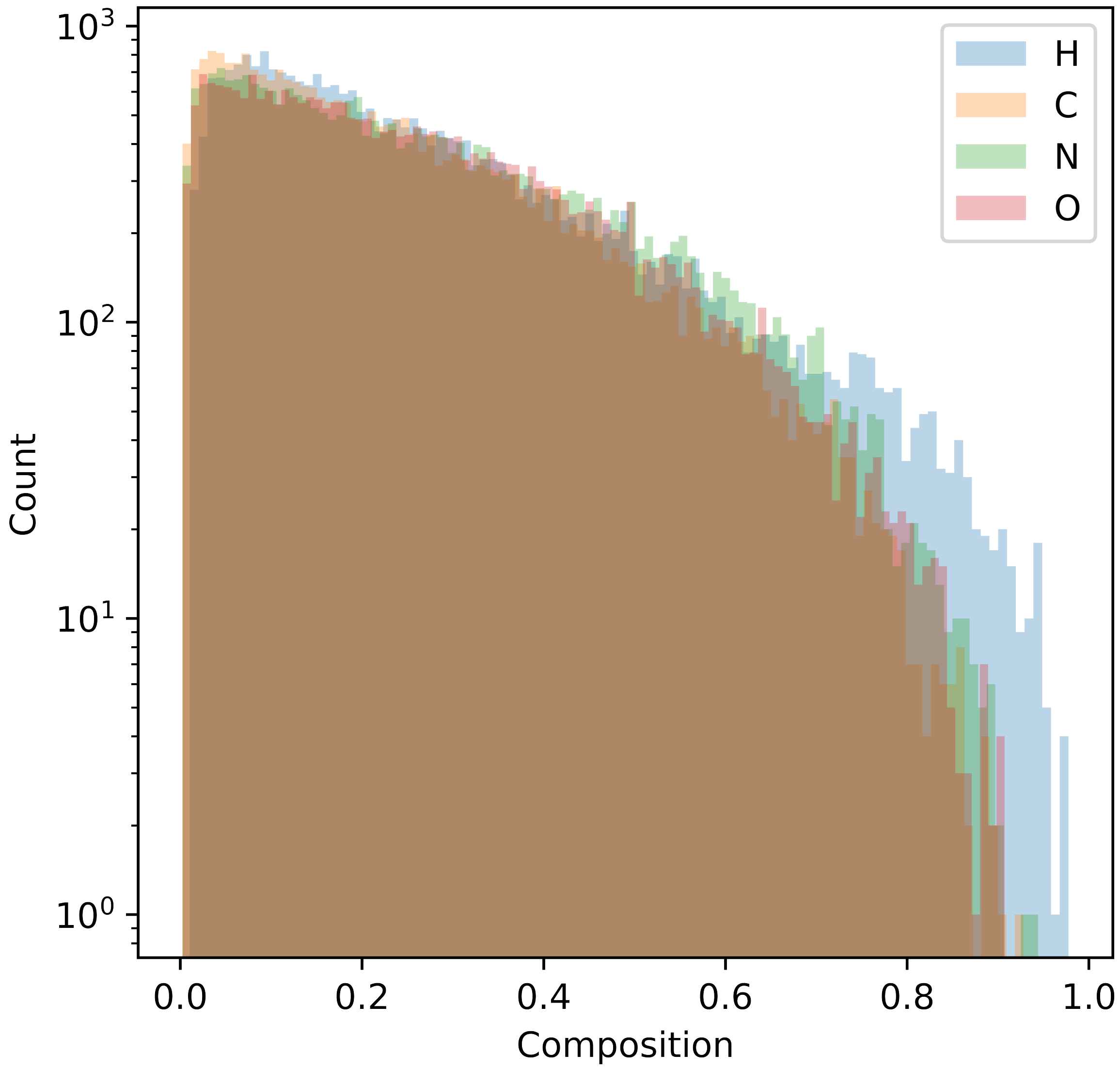

Figure 11. Histogram of the system composition of all systems in the training dataset, colored by element.

16 Mass density distribution of the ANI-1xnr training dataset

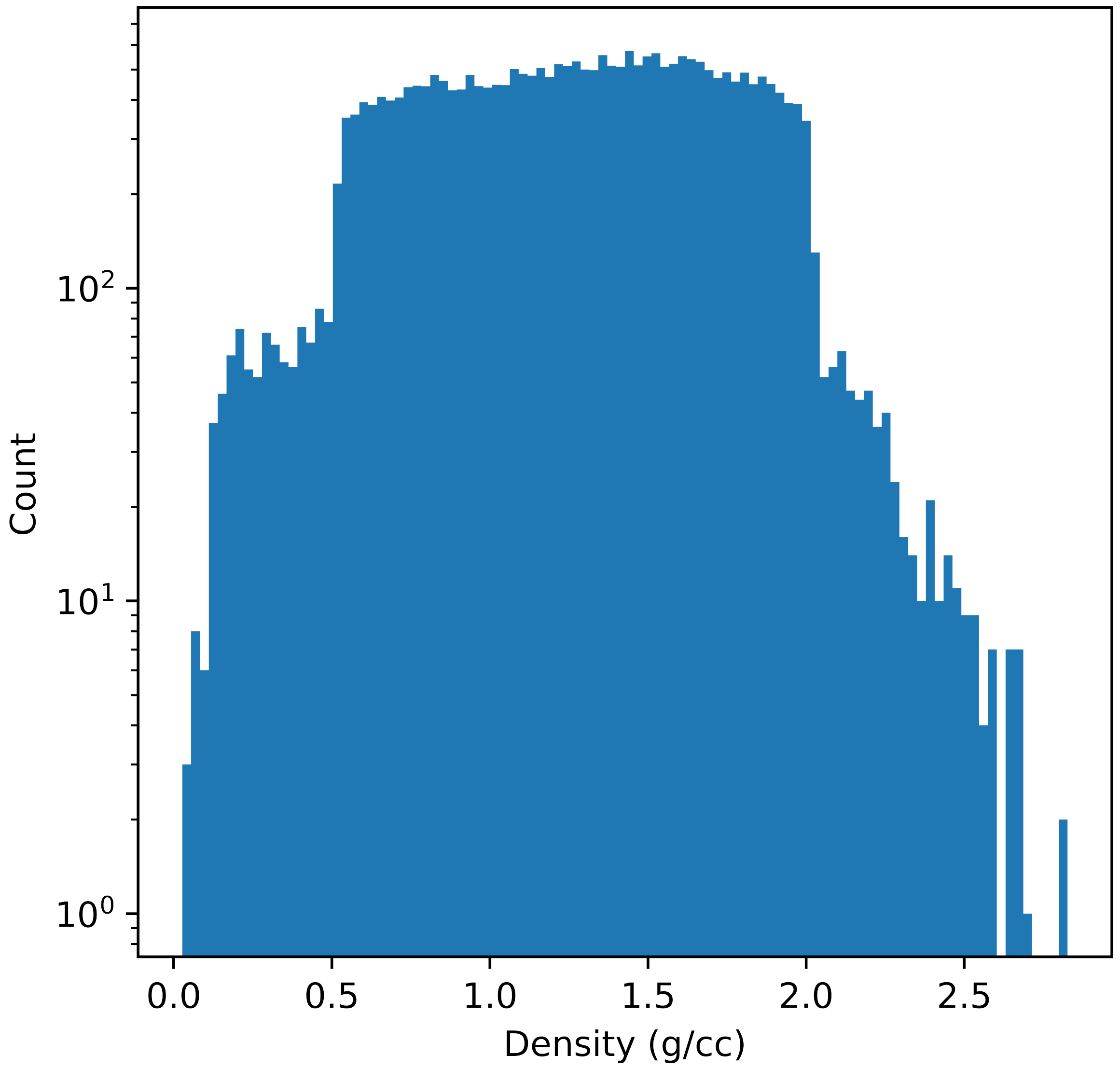

Figure 12. Histogram of the mass density (g/cc) of all systems in the training dataset.

17 Molecule size distribution of the ANI-1xnr training dataset

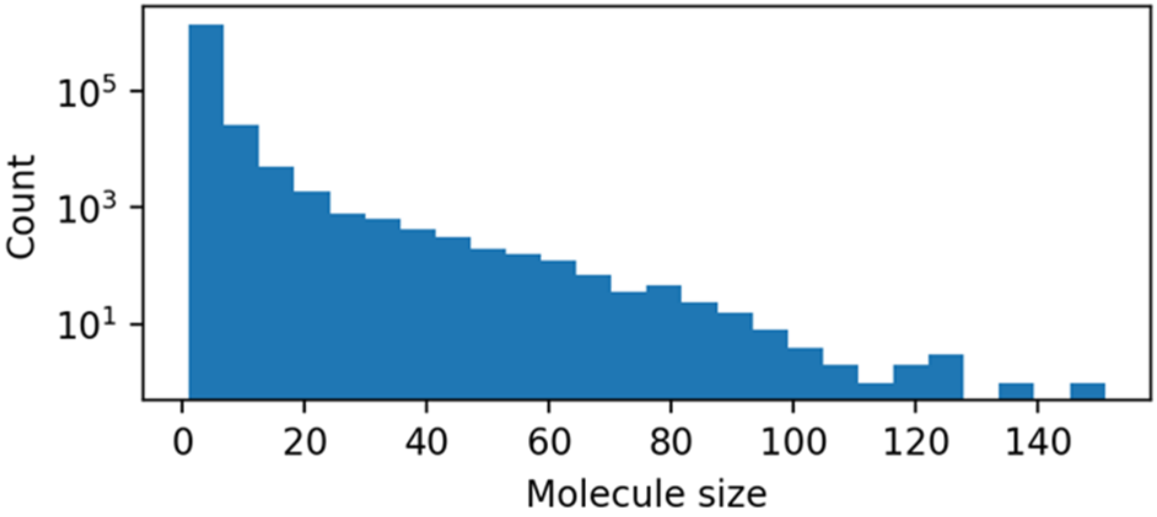

Figure 13. Distribution of the molecule size (i.e., number of heavy atoms) in the ANI-1xnr training dataset.

References

1. Kingma, D. P. & Ba, J. Adam: A Method for Stochastic Optimization, DOI: 10.48550/arXiv.1412.6980 (2017).
2. Montes de Oca Zapiain, D. *et al.* Training data selection for accuracy and transferability of interatomic potentials. *npj Comput. Mater.* **8**, 1–9, DOI: 10.1038/s41524-022-00872-x (2022).
